# Distributed processing for value-based choice by prelimbic circuits targeting anterior-posterior dorsal striatal subregions in male mice

**DOI:** 10.1038/s41467-023-36795-4

**Published:** 2023-04-06

**Authors:** Kyuhyun Choi, Eugenio Piasini, Edgar Díaz-Hernández, Luigim Vargas Cifuentes, Nathan T. Henderson, Elizabeth N. Holly, Manivannan Subramaniyan, Charles R. Gerfen, Marc V. Fuccillo

**Affiliations:** 1 Department of Neuroscience, Perelman School of Medicine, University of Pennsylvania, Philadelphia, PA USA; 2 Computational Neuroscience Initiative, University of Pennsylvania, Philadelphia, PA USA; 3 Neural Computation Lab, International School for Advanced Studies (SISSA), Trieste, Italy; 4 Neuroscience Graduate Group, Perelman School of Medicine, University of Pennsylvania, Philadelphia, PA USA; 5 Laboratory of Systems Neuroscience, National Institute of Mental Health (NIMH), Bethesda, MD USA

**Keywords:** Neural circuits, Reward

## Abstract

Fronto-striatal circuits have been implicated in cognitive control of behavioral output for social and appetitive rewards. The functional diversity of prefrontal cortical populations is strongly dependent on their synaptic targets, with control of motor output mediated by connectivity to dorsal striatum. Despite evidence for functional diversity along the anterior-posterior striatal axis, it is unclear how distinct fronto-striatal sub-circuits support value-based choice. Here we found segregated prefrontal populations defined by anterior/posterior dorsomedial striatal target. During a feedback-based 2-alternative choice task, single-photon imaging revealed circuit-specific representations of task-relevant information with prelimbic neurons targeting anterior DMS (PL::A-DMS) robustly modulated during choices and negative outcomes, while prelimbic neurons targeting posterior DMS (PL::P-DMS) encoded internal representations of value and positive outcomes contingent on prior choice. Consistent with this distributed coding, optogenetic inhibition of PL::A-DMS circuits strongly impacted choice monitoring and responses to negative outcomes while inhibition of PL::P-DMS impaired task engagement and strategies following positive outcomes. Together our data uncover PL populations engaged in distributed processing for value-based choice.

## Abstract (web summary)

The prelimbic region of the prefrontal cortex is involved in goal-directed action. Here the authors investigate the function of the prelimbic pathways projecting along the anterior posterior striatal axis in value based decision making in male mice.

## Introduction

Value-based decision-making requires a complex series of neural computations—the integration of success and failure, the proper attribution of actions to temporally displaced outcomes, and the monitoring of context and underlying task structure. One hypothesis posits that inputs for this decision-making process are represented across forebrain excitatory populations, with their integration in the striatum serving as an early step in action selection^1^. Consistent with a topographical organization of afferent inputs^2–4^, striatum exhibits functional segregation along its anatomical axes, with the dorsoventral divisions segregating reward and motor processes and medial-lateral domains supporting goal-sensitive and habitual action strategies^5^. However, less attention has been given to striatal function along the anterior-posterior (A-P) axis^6–10^, despite early retrograde studies pointing to a unique longitudinal (A-P) organization of cortical-striatal inputs^11^.

Seminal studies in rat provided the first evidence of functional segregation along the striatal A-P axis, with posterior dorsomedial striatum (P-DMS) lesions disrupting both the initial acquisition and post-training execution of instrumental conditioning, in particular modulation of response according to action-outcome association^8,9^. In contrast, the importance of the anterior dorsomedial striatum (A-DMS) in goal-directed choice remained uncertain, with opposing results for pharmacological inactivation and excitotoxic lesions^8,9,12^. Optogenetic manipulations of specific spiny projection neuron subtypes within the A-DMS have implicated this subregion in supporting flexible responses during reversal learning^13^, consistent with pharmacological manipulations of anterior caudate in marmosets^14^. In contrast, the anterior dorsolateral striatum (DLS) supports a protein synthesis-dependent consolidation of newly learned actions^15^. Finally, a growing body of evidence has implicated the rodent striatal tail, the most caudal subregion, in behavioral responses to aversive stimuli and psychostimulants^16–18^.

The prefrontal cortex exerts cognitive control over mammalian behavior via extensive afferent integration and widespread downstream connectivity^19,20^. Analysis of prefrontal populations accounting for downstream synaptic targets has revealed pathway-specific functional differences for prefrontal control of social-spatial rewards^21^, reward anticipation^22^, and choice directions^23^. The prelimbic region of the prefrontal cortex has been hypothesized to support goal-directed action by encoding short-term memories necessary for subsequent action-outcome associations in dorsal striatum^24^. Specific targeting of prelimbic-striatal pathways has extended this view, demonstrating persistent neural coding of value essential for choice behavior^25^ and the mediation of response inhibition during tasks requiring sustained attention^26^. Finally, DREADD-mediated inhibition of PL neurons projecting to either anterior or posterior striatal subregions has uncovered involvement in instrumental learning^6,10^.

Here we systematically explore the function of PL pathways projecting along the A-P striatal axis in male mice via integration of mono- and di-synaptic viral circuit tracing, local circuit connectivity measures, single neuron calcium imaging, statistical modeling of neural coding properties, and target-specific optogenetic manipulations. Retrograde tracing from A/P-DMS subregions revealed non-overlapping PL populations, which exhibited unique encoding of behavioral variables over multiple time scales essential for shaping efficient action selection and execution. Target- and temporally- specific optogenetic manipulations confirmed the functional divergence of these fronto-striatal pathways, with PL::A-DMS pathways supporting choice monitoring and responding to negative outcomes and PL::P-DMS pathways supporting task motivation and responding to positive outcomes. Together, our results provide insight into the distributed nature of fronto-striatal pathways for decision making.

## Results

### Anatomical architecture of fronto-striatal pathways along the anterior-posterior striatal axis

**Fig. 1:**
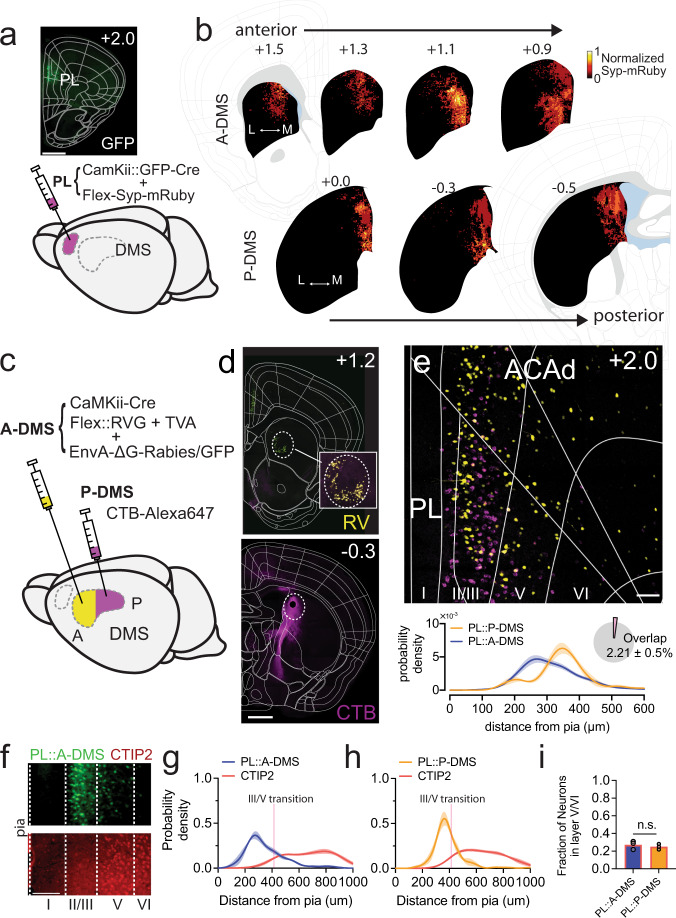
Distinct PL neuron populations defined by anterior/posterior dorsomedial striatal (DMS) target. **a** Approach for anterograde tracing of PL-DMS excitatory projections with synaptic terminal marker Synaptophysin-mRuby (inset shows GFP-Cre expression at PL target site, scale bar, 1000μm, *n* = 4 animals). **b** Averaged fluorescent intensity of Synaptophysin-mRuby inputs from PL along anterior-posterior axis, superimposed onto striatum in brain atlas (top: A-DMS, bottom: P-DMS, left to right increasingly posterior, *n* = 4 animals). Top number denotes A/P coordinates from bregma (mm). **c** Schematic demonstrating dual retrograde tracing strategy using trans-synaptic rabies virus (A-DMS; yellow) and Alexa647-conjugated CTB (P-DMS; magenta). **d** Coronal sections showing injection sites (top: A-DMS, Bottom: P-DMS, *n* = 5 animals). scale bar, 1000 μm. Number in upper right corner denotes A/P coordinate from bregma. **e** Representative image of medial prefrontal cortex (*top*) and quantification (kernel density estimate) of neuronal density from the pia (*bottom*, *n* = 4 animals), with relative proportion of overlapping double-labeled neurons (*inset*). scale bar, 100 μm. **f** Example image showing prelimbic area from EnvA-∆G-rabies virus tracing of A-DMS inputs (*top*) co-stained with CTIP2 (*bottom*, *n* = 3 animals). Scale bar 100 μm. **g** Quantification of neuronal density from pia of PL::A-DMS and CTIP2+ populations (*n* = 3 animals). Solid line, mean; shaded area, ± SEM. **h** Quantification of neuronal density from pia of PL::P-DMS and CTIP2 + populations (*n* = 3 animals). Pink line in g, h represents average layer III-V transition as visualized by increased density of CTIP2 + neurons in layer V. Solid line, mean; shaded area, ± SEM. i) Fraction(mean ± SEM) of GFP labeled neurons located in CTIP2 + deep cortical layers (*p* = 0.54, *n* = 3/3 animals, Two-sided unpaired t-test).

To explore whether distinct afferent connectivity could explain previously described differences in DMS function along the anterior-posterior axis^8,27^, we injected two distinct Alexa-conjugated Cholera toxin subunit-B retrograde tracers into A-DMS and P-DMS (Fig. S1a). Excepting the amygdala, most afferent projection regions targeted both DMS striatal areas with non-overlapping neuronal populations (Fig. S1j). To better understand the functional implications of this unique circuit architecture, we focused on prefrontal cortical areas, particularly prelimbic cortex (PL), which despite a bias towards A-DMS, targeted both DMS compartments (Fig. S1j). To confirm that synaptic inputs from PL were spread along the full anterior-posterior extent of DMS, we injected a mix of AAV5-CamKII::GFP-Cre and AAVdj-EF1a::Flex-Synaptophysin-mRuby virus into PL (Fig. 1a, b).

To address whether these widespread projections arose from *en passant* connectivity or distinct PL afferents, we utilized two orthogonal retrograde circuit tracers, with EnvA G-deleted rabies virus EGFP injected in A-DMS, and Alexa647-conjugated Cholera toxin subunit-B (CTB) injected in P-DMS (Fig. 1c). This design minimized fiber-of-passage contamination of PL::P-DMS pathways while traversing A-DMS (see Methods for details). Using CTIP2 immunostaining as a marker of cortical deep layers^28^, we found cell bodies of both retrogradely labeled populations largely in prelimbic layers II/III and more sparsely in layers V/VI (Fig. 1f–i). Regardless of layer, these populations were distinct (only 2.2 ± 0.5% overlap) and spatially separated, forming a characteristic sub-layer structure with PL::A-DMS populations localized to superficial layer II/III and PL::P-DMS populations found in deeper layer II/III (Fig. 1e).

### PL::A/P-DMS circuits are characterized by biased afferent drivers and distinct local striatal synaptic connectivity

**Fig. 2:**
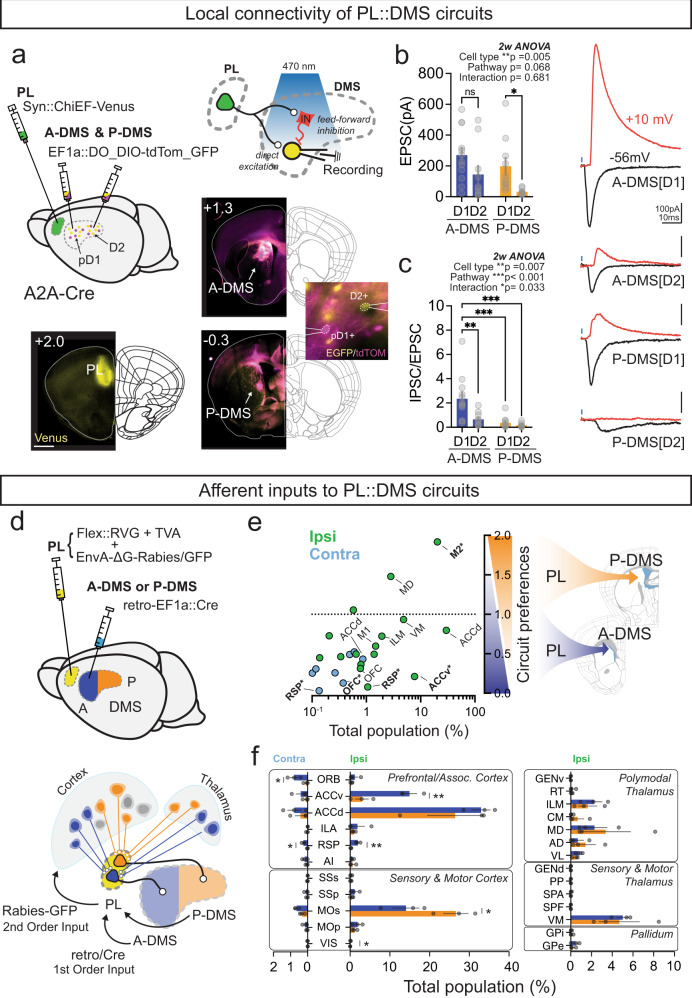
Characterizing afferent drivers and local striatal connectivity of PL::A/P-DMS circuits. **a** Schematic of experiment to examine local connectivity of PL::DMS using channelrhodopsin variant (ChiEF; PL) and cell type marker (DO/DIO-GFP/tdTomato; A and P-DMS) in Adora2a-Cre mice. (Insets show viral expression at 3 representative target sites, scale bar, 1000 μm). (Top-right) Schematic of synaptic wiring between PL::DMS. **b**, **c** Average amplitude (mean ± SEM) of direct excitatory synaptic current (*top-left*), Excitatory/Inhibitory ratio (*bottom-left*, mean ± SEM) depends on target DMS area and cell type. Representative traces from each group (*right)*. Black trace, monosynaptic excitatory current; red trace, feed-forward inhibitory current. Šidák multiple comparison test, *p < 0.05, **p < 0.01, ***p < 0.001. (A-DMS[D1]/A-DMS[D2]/P-DMS[D1]/P-DMS[D2] *n* =  12/10/10/9 cells from five independent animals). **d** Schematic of tracing approach to label 2nd order projections to PL circuits defined by DMS target. RetroAAV2-EF1a::Cre virus was injected into either A/P-DMS with Cre-sensitive TVA receptor and Rabies virus glycoprotein injected separately into PL, followed by EnvA pseudo typed-∆G-Rabies virus one week later. **e** Brain-wide innervation preferences of PL::A/P DMS pathways. Abscissa shows relative proportion (out of total labeled neurons) for each brain region and ordinate shows the ratio between pathways (PL::P-DMS/PL::A-DMS). Green and blue circles represent ipsilateral and contralateral sites relative to injection. *p < 0.05, **p < 0.01. **f** Comparison of second-order innervation (mean ± SEM) from major afferent brain areas (ORB^contra^ *p* = 0.0406; ACAv^ipsi^ *p* = 0.008; RSP^contra^ *p* = 0.010, RSP^ipsi^ *p* =0.008; MOs^ipsi^ *p* = 0.0497; VIS^ipsi^ *p* = 0.023; *n* = 3/3 animals/each group, Two-sided unpaired t-test). *p < 0.05, **p < 0.01.

To begin exploring potential functional differences of these PL-DMS circuits, we examined their specific synaptic connectivity within anterior and posterior DMS (Fig. 2a–c). We injected AAVdj-Syn::ChiEF-2a-Venus into PL cortex and AAVdj-EF1a::DO/DIO-GFP/tdTomato virus into A/P-DMS in A2A-Cre mice, permitting simultaneous labeling of the direct (dSPN, Cre-, tdTOM+) and indirect (iSPN, Cre+, GFP+) spiny projection neuron subtypes. We used 470 nm light to recruit PL synaptic terminals in striatum while patching identified SPNs in voltage clamp configuration, holding neurons sequentially at −56mV and +10 mV, to specifically isolate direct glutamatergic and di-synaptic feed-forward GABAergic currents, respectively (Fig. 2a–c). We found that dSPNs in both circuits received stronger excitatory synaptic inputs than iSPNs but didn’t observe differences in dSPN excitatory drive depending on A-P DMS target (Fig. 2b). We next measured the amount of feed-forward inhibition—confirmed by sensitivity of light-evoked GABAergic currents to AMPAR block (Fig. S2)—normalized to the monosynaptic excitatory current. Interestingly, we found that PL connections to A-DMS dSPNs recruited a substantially larger feed-forward inhibition than all other connections (Fig. 2c).

Beyond postsynaptic connectivity, another source of circuit diversity lies in the inputs that neurons receive. To investigate this, we examined second-order connectomes for PL neurons defined by A/P-DMS subregion by injecting retroAAV2-EF1a::3xFLAG-Cre into either A- or P-DMS subregions and a mixture of AAV-DJ-CAG::FLEX-TVA-mCherry and AAV-DJ-CAG::DIO-RVG into PL cortex (Fig. 2d). Subsequent PL injection of EnvA-RV-EGFP permitted single synapse tracing specifically from PL neurons that projected to either DMS subregion (2nd order inputs). Consistent with these fronto-striatal circuits being embedded in the same local microcircuit, we observed multiple afferent populations with similar targeting of each PL circuit, including dorsal anterior cingulate cortex (dACC) and both associative and ventral motor thalamic nuclei (Fig. 2e, f). Surprisingly though, we also noted pathway-specific distinctions in second order afferent connections, with strong PL::P-DMS biases from secondary motor cortex (M2) and significant PL::A-DMS biases from ventral anterior cingulate cortex, retrosplenial cortex and orbitofrontal cortex. Together, these data show that distinct PL populations determined by A/P-DMS target have distinct striatal synaptic connectivity and biases in their afferent drivers.

### Assessing neural activity in PL::DMS pathways during a value-based choice task

**Fig. 3:**
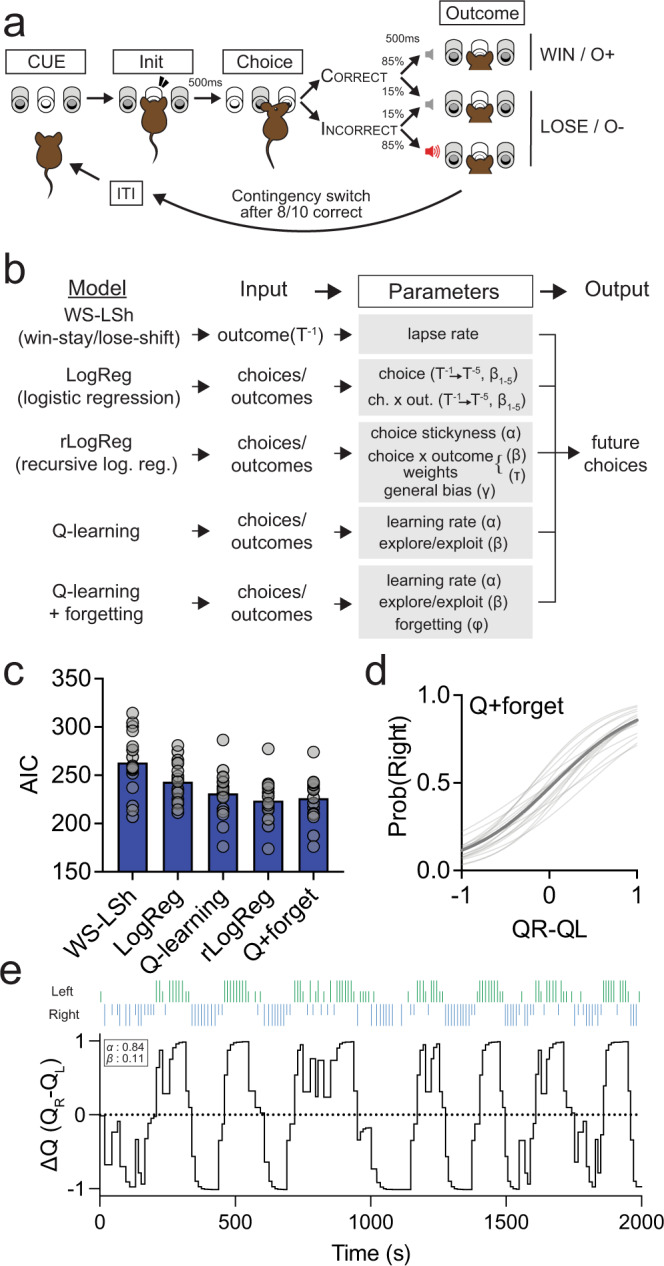
Quantifying behavior models of a value-based choice task. **a** Schematic of trial structure showing mice initiating trials via sustained (500 ms) center port entry, followed by left/right choice within 3 s. Subsequent reward is delivered from center port. **b** Five behavior models using choice and reward history to predict current animal choice. **c** AIC comparison (mean ± SEM) from five behavior models of choice behavior (WS-LSh, WinStay-LoseShift; LogReg, Logistic Regression; *Q*-learning, standard q-learning model; rLogReg, recursive Logistic Regression; *Q*+forget, q-learning model with forgetting for unchosen choice). **d** Relationship between the probability of right choice and ∆*Q* value obtained from *Q*+forget model. Mean (thick gray line) and individual animal replicates (thin gray line). **e** Trial-by-trial choices (blue bar, right; green bar, left), outcomes (long bar, positive; short bar, negative) and predicted ∆Q-values derived from *Q* + forget model.

The distinct afferent connectivity of these PL::DMS circuits suggests they may support divergent neural processes for the control of action selection. To explore this possibility, we investigated neural coding of task-relevant information within PL::A-DMS and PL::P-DMS populations during a value-based choice task. Mice were trained on a 3-poke chamber where the center port initiated a choice period, requiring a lateral left/right decision. In any given trial, choosing a predetermined side led to the delivery of a reward with 85% chance and no outcome otherwise, while choosing the opposite port led to punishment tone with 85% chance and no outcome otherwise (Fig. 3a). The identity of the rewarded side (or “contingency”) was changed whenever mice made 8 correct choices over the latest 10 trials, to assess flexible responding. Viral overexpression of Kir2.1, which strongly suppresses neuronal activity (Fig. S3k–p), in SPNs of either A-DMS or P-DMS (Fig. S3a,d) provided initial evidence that these striatal territories distinctly contributed to performance in this task (Fig. S3b,e), with A-DMS silencing increasing lose-stay choice (Fig. S3c) and silencing of both areas decreasing win-stay choice (Fig. S3c, f). Notably, overexpression of Kir2.1 in A-DMS led to increased acquisition rate in instrumental learning, while in P-DMS it led to devaluation resistance (Fig. S3g–j).

To estimate key latent variables that shape choice strategies in our task, we fit five behavioral models (Fig. 3b, see “Methods” for specific model details) and compared their ability to parsimoniously explain choice data (Figs. 3c, S4d). A simple win-stay/lose-shift model performed worst, consistent with the near-random choice patterns exhibited by mice following unrewarded prior outcomes (Fig. S4a). We also used a logistic regression (LogReg) of prior choice and reward history (Fig. S4b) as well as a recursive logistic regression (rLogReg) model that integrates prior choice “stickiness” and estimates of being in left versus right rewarded port state (Fig. 3b; Fig. S4c)^29^. Finally, we used two versions of *Q*-value reinforcement learning (RL) models, wherein values for each choice are incrementally updated in a trial-by-trial manner (Fig. 3d, e). As judged by standard model comparison metrics (Fig. 3c; Fig. S4d), we found that the rLogReg and Q-learning RL model with forgetting (*Q*+forget) provided a similar optimal mix of predictive power for given free parameters. Consistent with this, we found a strong correlation between the state estimates of the rLogReg model and the difference in *Q* values (Δ*Q*) of the RL model (Fig. S4e, f). We hereafter used the *Q*-value RL model with forgetting, as it provided the most intuitive trial-by-trial value estimates (Fig. 3e) and has been used previously in similar analyses^25,30^.

**Fig. 4:**
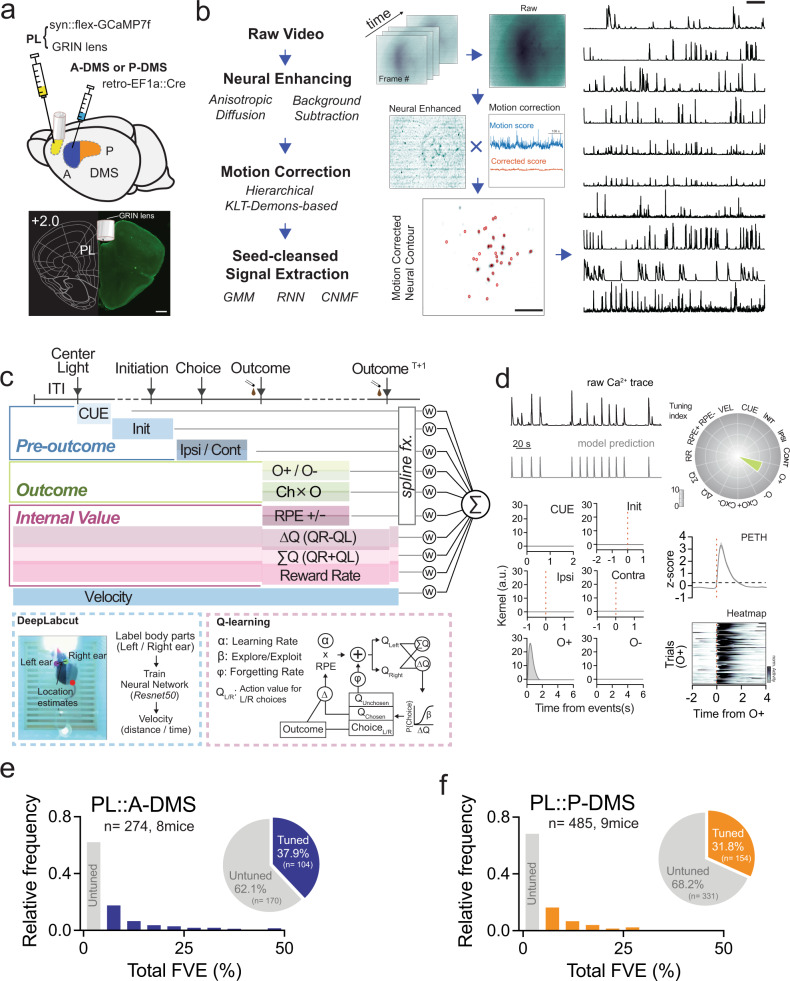
Assembly of neural encoding model during operant task with 1-photon imaging. **a** Schematic showing viral injection strategy to label pathway specific PL neurons for 1-photon calcium imaging (*top*) and representative image indicating GRIN lens location (*bottom*, *n* = 17 animals). scale bar, 500 μm. **b** MIN1PIPE workflow (*left*) for extraction of calcium signal and snapshot for each step (*middle*). scale bar, 50 pixels. Representative raw Ca^2+^ traces from 10 neurons (*right*). Scale bar, 20 s. **c** Abstract of design matrix structure for neural encoding model showing behavioral predictors for pre-outcome, outcome, internal representations of value (*top*, see Table S1). Schematic of DeepLabcut pipeline for estimating head velocity (blue box) and *Q* + forget modeling (pink box) to estimate latent internal choice values *(bottom)*. **d** Example of raw Ca^2+^ trace and predicted trace from full encoding model (*top, left*). Encoding model inferred kernels (*bottom, left*) from example neuron exhibiting strong O+ modulation (*top, right*, tuning plot). Radar plot shows overview of tuning indices from representative neuron. Peri-event time histogram and trial-by-trial neuronal activity heat map (*bottom, right*) aligned by O+. e, f) Histogram of binned total FVE distribution for all neurons from PL::A-DMS (**e**) or PL::P-DMS (**f**). Gray bars denote non-task tuned population (<5% FVE threshold); colored bars (blue, PL::A-DMS; orange, PL::P-DMS) denote task-tuned neurons. Pie charts showing the proportion of task-tuned neurons (insets).

We performed 1-photon (1-p) single neuron calcium (Ca^2+^) imaging of retrogradely-labeled PL neurons expressing GCaMP7f during this task. Given the minimal fiber-of-passage overlap with standard retrograde tracers (Fig. S1a–c), we injected retroAAV2-EF1a::3XFLAG-Cre into either A-DMS or P-DMS, together with AAV1-hSyn::FLEX-jGcamp7f into PL to gain access to both PL populations in separate animals (Fig. 4a). Using this approach and the MIN1PIPE 1-p signal analysis pipeline (Fig. 4b)^31^, we recorded Ca^2+^ activity of 274 PL::A-DMS neurons and 485 PL::P-DMS neurons. To analyze neural activity, we designed a linear encoding model based upon task-relevant regression predictors capturing pre-outcome activity, resulting outcomes, and RL model-based estimations of internal value (Fig. 4c). Pre-outcome variables included trial start cue (cue), self-initiation poke (Init) and Ipsilateral/Contralateral (Ipsi/Cont) choice, defined as the choice side in relation to the recording site. Outcomes were divided into positive (O+) and negative (O−), as well as interactions of these terms with prior choice, a relevant neural signal for credit assignment (Ch x O+, Ch x O−). Latent estimations of choice values inferred from the Q-learning scheme were included in the neural encoding model as predictors representing trial-by-trial differences in choice value (Δ*Q*), local reward environment (ΣQ) and positive/negative reward prediction errors (RPE+/−) (Figs. 4c, 3e). Local reward rate over the last 5 trials was included as a proxy for task engagement. We also included head velocity extracted from marker-less pose-capture analyses (Fig. 4c, see “Methods”) to account for neural representations of movement^32^. We excluded head acceleration as a proxy of motor vigor because it did not further improve model fits (Fig. S5c, d). Regression parameters were fit via elastic-net penalized maximum likelihood (see “Methods” for details on model design and fitting).

We applied this encoding model to both PL::A-DMS and PL::P-DMS Ca^2+^ imaging data, measuring total model fit quality by calculating the fraction of Ca^2+^ signal variance explained (FVE). At a cut-off threshold of 5% FVE, our model fit ~38% of total PL::A-DMS neurons and ~32% of total PL::P-DMS neurons (Fig. 4e, f). To quantify neuronal tuning to specific behavioral variables, partial models lacking the related predictors were fit to Ca^2+^ data. The difference in FVE between the full and the partial model defined a lower-bound for tuning to given variables (Fig. S5a). Changes of FVE threshold did not change PL pathway-specific population tuning noted below (Fig. S5e, f).

### PL::A-DMS and PL::P-DMS neural populations encode distinct and complementary components of value-based choice behavior

**Fig. 5:**
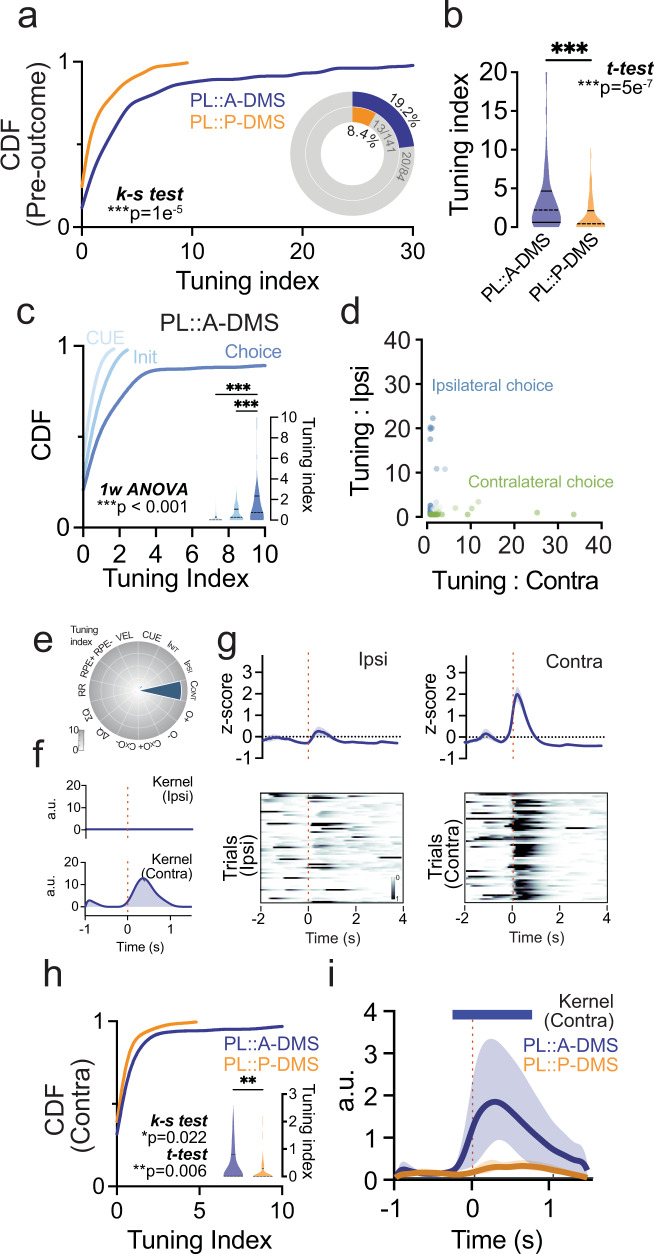
Pre-outcome tuning is dominated by preferential encoding of choice in PL::A-DMS. **a** Comparison of cumulative distribution of pre-outcome group tuning (*p* = 1e−5, Two-sided Kolmogorov-Smirnov test) and the proportion of highly tuned neurons to pre-outcome group predictor (*insets*, >5% tuning index). ***p < 0.001. **b** Comparison of average tuning index(Shaded area, Kernel probability density; solid line, quartile=0.4965,4.571/0,2.028; dotted line, median = 2.211/0.4421) between PL::A- or P-DMS for pre-outcome predictors. (*p* = 5e−7, PL::A-DMS, *n* = 104 cells; PL::P-DMS, *n* = 154 cells, Two-sided unpaired t-test). ***p < 0.001. **c** Comparison of cumulative distributions of individual components for pre-outcome group predictors from PL::A-DMS and average tuning index (insets; Shaded area, Kernel probability density; solid line, quartile; dotted line = 0.0.2959/0,1.057/0.2.334, median = 0/0.2718/0.7517; *n* = 104 cells, CUE-Init, *p* = 0.86; CUE-Choice, *p* = 8e−6; Init-Choice, *p* = 2e−7, Šidák multiple comparison test). ***p < 0.001. **d** Scatter plot of Ipsi/Contra tuning index from task tuned neurons (green: Contra- encoding neurons, blue: Ipsi-encoding neurons). **e** Tuning plot showing representative contralateral choice tuned neuron from PL::A-DMS. **f** Encoding model inferred kernels corresponding to ipsi (*top*) and contra (*bottom*) choice. **g** PETH (*top*) and trial-by-trial neuronal activity (*bottom*) aligned by Ipsi (*left*)/Contra (*right*) choice. Solid line, mean; shaded area, ±SEM. **h** Comparison of cumulative distribution(*p* = 0.022, Two-sided Kolmogorov-Smirnov test) and average tuning index (insets; Shaded area, Kernel probability density; solid line, quartile = 0.0.7890/0,0.3020; dotted line, median = 0.0101/0) of contra choice tuned neurons in both PL::A/P-DMS pathways (*p* = 0.006, PL::A-DMS, *n* = 104 cells; PL::P-DMS, *n* = 154 cells, Two-sided unpaired t-test). **p < 0.01. **i** Comparison of model inferred contralateral choice kernels on average for both PL::DMS pathways. Solid line, root-mean-squared (RMS); shaded area, ±95% confidence interval. Colored bar on top indicates timepoints for which RMS kernels are significantly different between pathways (bootstrap test).

To broadly assess pathway-specific PL tuning to events preceding outcome feedback, we grouped the cue, initiation, and choice predictors. We found that PL::A-DMS circuits were more strongly tuned to these pre-outcome events (Fig. 5a,b), with the majority of modulation driven by choice-associated tuning (Fig. 5c). In our encoding model, choice-associated activity may reflect components of action selection (decision process, motor command, ongoing motor kinematics) or choice evaluation (predictive or efference signals). We found that PL::A-DMS encoded both ipsilateral and contralateral choices (Fig. S6a, b) in a near exclusive manner (Fig. 5d). Despite the PL::A-DMS pathway bias for generally encoding movement kinematics (Fig. S6h), we found that the majority of choice modulated neurons did not strongly encode head velocity (Fig. 5e and Fig. S6i), nor the specific turning actions required to enter ports (Fig. S6j).

Our tuning index is a compact measure of the degree to which task-related variables are represented in neural activity. Nonetheless, it only captures overall coding strength and is not sensitive to the precise temporal evolution of neural responses, which could help attribute choice signals to action selection or evaluation processes. To address this, we analyzed the event-associated kernels inferred by our encoding model, which estimate the average calcium activity transient elicited by specific behavioral events, after accounting for overlapping transients from other event types (see “Methods”). Analysis of choice-associated kernels revealed that PL::A-DMS neurons exhibited robust activity starting just prior (~250 ms) to choice execution (Figs. 5i, S6g) and these kernels were on average stronger for choices contralateral to the recording site (Fig. S6b). These data suggest a sub-population of PL::A-DMS neurons encode either direct motor commands or evaluative signals for future choice.

**Fig. 6:**
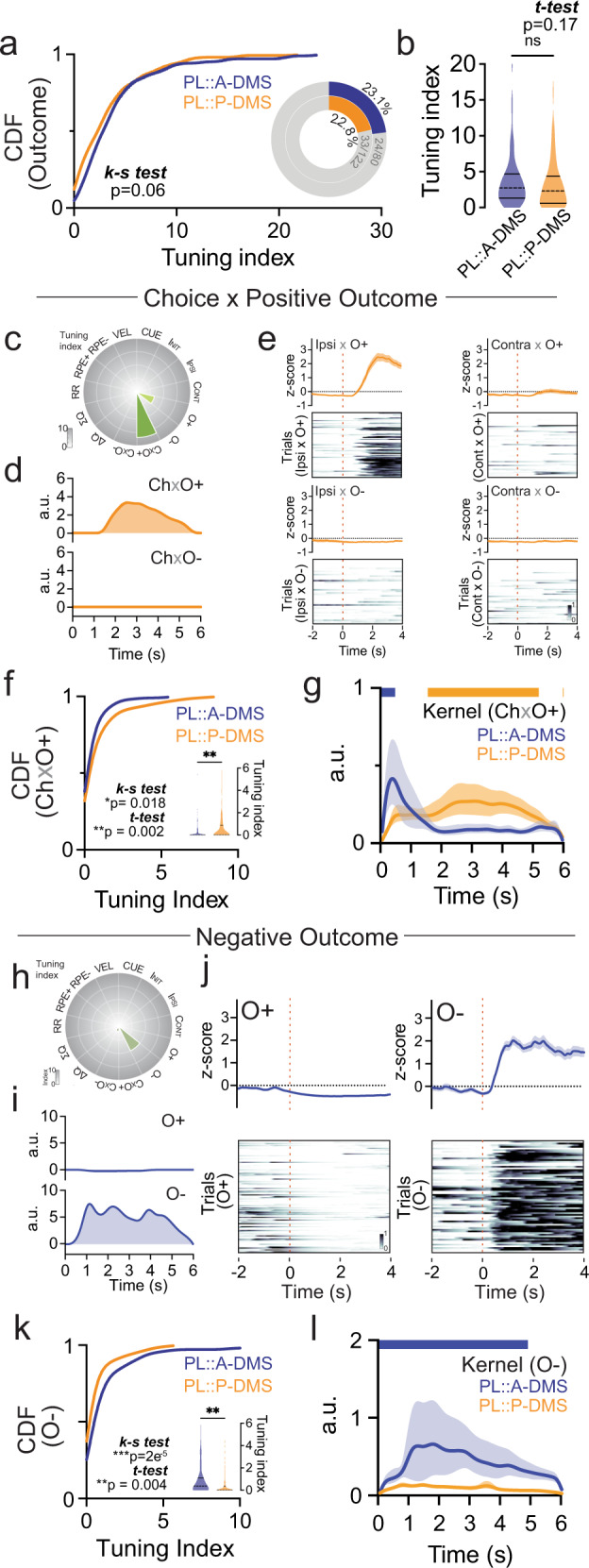
Divergent encoding of outcome by PL::DMS pathways. **a** Comparison of cumulative distribution of outcome group tuning index (*p* = 0.06, Two-sided Kolmogorov–Smirnov test) and the proportion of highly tuned neurons to outcome group predictor (*insets*, >5% tuning index). **b** Comparison of average tuning index (Shaded area, Kernel probability density; solid line, quartile = 1.309,4.695/0.5896,4.394; dotted line, median = 2.695/2.318) between PL::A- or P-DMS for outcome predictors (*p* = 0.17, PL::A-DMS, *n* = 104 cells; PL::P-DMS, *n* = 154 cells, Two-sided unpaired t-test). **c** Tuning plot showing representative Ch x O+ tuned neuron from PL::P-DMS. **d** Encoding model inferred kernels corresponding to Ch x O+ (*top*) and Ch x O- (*bottom*). **e** Four interactions of PETH (*top*) and trial-by-trial neuronal activity (*bottom*) aligned by outcome. Solid line, mean; shaded area, ±SEM. **f** Comparison of cumulative distribution (*p* = 0.02, Two-sided Kolmogorov-Smirnov test) and average tuning index (insets; Shaded area, Kernel probability density; solid line, quartile = 0, 0.2009/0, 0.8864; dotted line, median = 0, 0) of Ch x O+ tuned neurons in both PL-A/P-DMS pathways (*p* = 0.002, PL::A-DMS, *n* = 104 cells; PL::P-DMS, *n* = 154 cells, Two-sided unpaired t-test). *p < 0.05, **p < 0.01. **g** Comparison of model inferred Ch x O+ kernels on average for both PL-DMS pathways. Solid line, RMS; shaded area, ±95% confidence interval. Colored bar on top indicates timepoints for which RMS kernels are significantly different between pathways (bootstrap test). **h** Tuning plot showing representative O- tuned neuron from PL::A-DMS. **i** Encoding model inferred kernels corresponding to O+ (*top*) and O− (*bottom*). **j** PETH (*top*) and trial-by-trial neuronal activity (*bottom*) aligned by outcome. Solid line, mean; shaded area, ±SEM. **k** Comparison of cumulative distribution (*p* = 2e−5, Two-sided Kolmogorov–Smirnov test) and average tuning index (insets; Shaded area, Kernel probability density; solid line, quartile = 0,1.107/0,0.3613; dotted line, median=0.3689/0) of O- tuned neurons in both PL-A/P-DMS pathways (*p* = 0.004, PL::A-DMS, *n* = 104 cells; PL::P-DMS, *n* = 154 cells, Two-sided unpaired t-test). **p < 0.01, ***p < 0.001. **l** Comparison of model inferred O- kernels on average for both PL-DMS pathways. Solid line, RMS; shaded area, ±95% confidence interval. Colored bar on top indicates timepoints for which RMS kernels are significantly different between pathways (bootstrap test).

Next, we examined how distinct PL::A/P-DMS pathways responded during behavioral outcomes. While broadly defined outcome tuning was seen in a similar proportion of neurons in each circuit (Fig. 6a, b), we found strong pathway specific biases dependent on the associated valence and time course of neural signals. One common feature of both PL::A/P-DMS pathways was a brief (~1 s) response immediately following all positive outcomes that was similar in waveform kinetics (Fig. S7a–h). In contrast, we found that PL::P-DMS circuits more strongly encoded positive outcomes contingent on prior choice (Ch x O+; Fig. 6c–g) than PL::A-DMS populations (Fig. 6f). Furthermore, the temporal kinetics of these interaction signals were distinct between pathways, with activity in PL::P-DMS pathways growing and persisting for several seconds beyond outcome, as compared to briefer Ch x O + signals in PL::A-DMS neurons (Fig. 6g). Finally, we observed robust neuronal responses to negative outcomes that were encoded strongly by PL::A-DMS neurons (Fig. 6h–l). These signals exhibited a slow and persistent increase following the absence of reward, which occurred at contingency switches, random unrewarded trials or during brief exploratory choice periods (Fig. 6j, l). Together, these data reveal a distributed representation of outcomes by PL::DMS pathways, with prolonged activation of PL::P-DMS neurons encoding positive outcomes contingent on prior choice and PL::A-DMS neurons encoding negative outcomes.

**Fig. 7:**
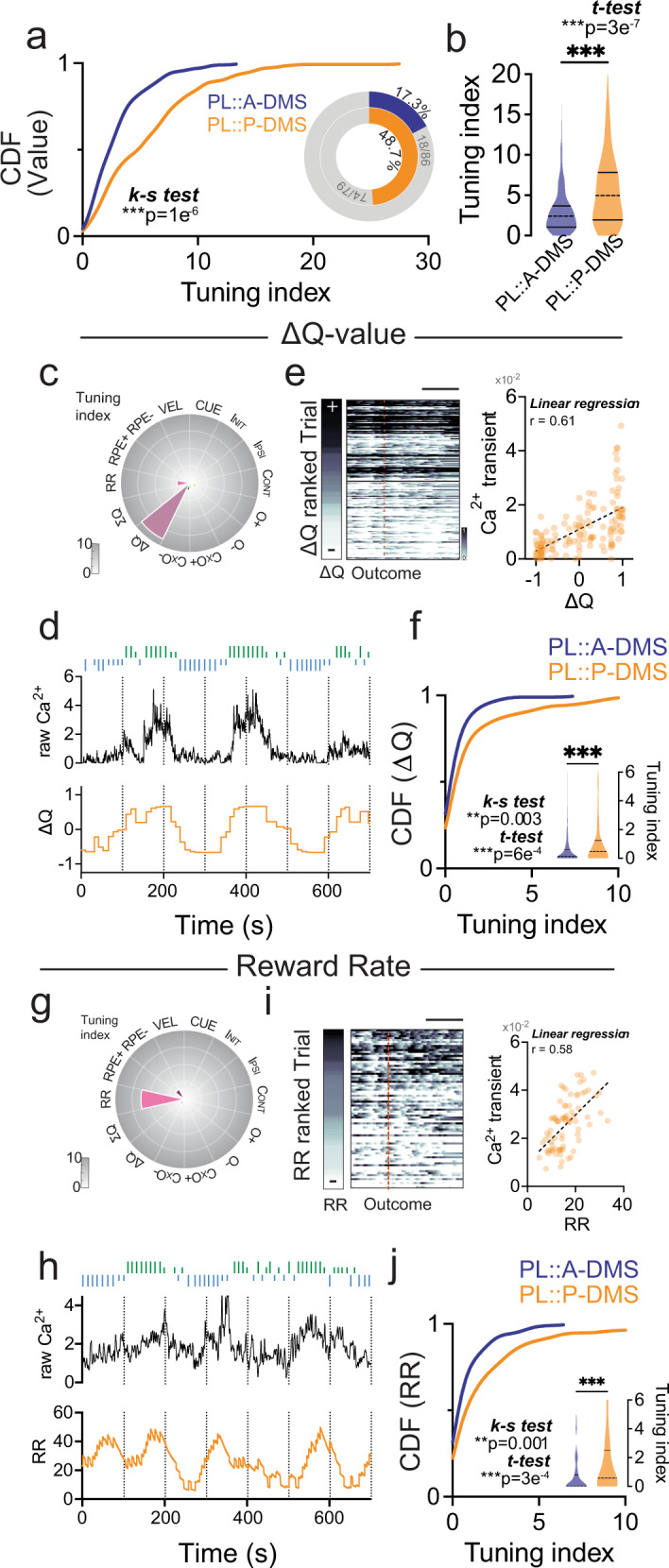
Preferential representation of internal value in PL::P-DMS. **a** Comparison of cumulative distribution of internal value tuning index (*p* = 1e−06, Two-sided Kolmogorov–Smirnov test) and the proportion of highly tuned neurons to internal value predictor (*insets*, >5% tuning index). **p < 0.01. **b** Comparison of average tuning index (Shaded area, Kernel probability density; solid line, quartile = 1.052,3.685/1.939,7.831; dotted line, median = 2.410/4.972) between PL::A- or P-DMS for internal value predictors (*p* = 3e−7, PL::A-DMS, *n* = 104 cells; PL::P-DMS, *n* = 154 cells, Two-sided unpaired t-test). ***p < 0.001. **c** Tuning plot showing representative ∆*Q* tuned neuron from PL::P-DMS. **d** Time course of raw Ca^2+^ (black, *top*) and ∆*Q* (orange, *bottom*) trace. Choices and outcomes at top (left/right, green/blue bar; O+/O−, long/short bar). **e** Outcome aligned trial-by-trial transient Ca^2+^ signals ranked by ∆*Q* (*left*; scale bar, 2 s) and scatter plot showing linear correlation between ∆*Q* and trial average of Ca^2+^ transients (*right*, orange dots denote single trial for a given ∆*Q* and Ca^2+^ signals, black dotted line from linear regression. *r* = 0.61, *p* < 1e−12, *n* = 135 trials, simple linear regression). **f** Comparison of cumulative distribution (*p* = 0.003, Two-sided Kolmogorov–Smirnov test) and average tuning index (insets; shaded area, Kernel probability density; solid line, quartile = 0,0.5914/0,1.239; dotted line, median = 0.1281/0.4622) of ∆*Q* tuned neurons in both PL-A/P-DMS pathways (*p* = 6e−4, PL::A-DMS, *n* = 104 cells; PL::P-DMS, *n* = 154 cells, Two-sided unpaired t-test). **p < 0.01, ***p < 0.001. **g** Tuning plot showing representative RR tuned neuron from PL::P-DMS. **h** Time course of raw Ca^2+^ (black, *top*) and RR (orange, *bottom*). Choices and outcomes at top (left/right, green/blue bar; O+/O−, long/short bar). **i** Outcome aligned trial-by-trial transient Ca^2+^ signals ranked by RR (*left*; scale bar, 2 s) and scatter plot showing linear correlation between RR and trial average of Ca^2+^ transients (*right*). Orange dots denote single trial for a given RR and Ca^2+^ signals, black dotted line from linear regression (*r* = 0.58, *p* < 1e−12, *n* = 79 trials, simple linear regression). **j** Comparison of cumulative distribution (*p* = 0.0012, Two-sided Kolmogorov–Smirnov test) and average tuning index (insets; Shaded area, Kernel probability density; solid line, quartile = 0,0.7863/0,2.503; dotted line, median = 0.009/0.5833) of RR tuned neurons in both PL-A/P-DMS pathways (*p* = 3e−4, PL::A-DMS, *n* = 104 cells; PL::P-DMS, *n* = 154 cells, Two-sided unpaired t-test). **p < 0.01, ***p < 0.001.

Finally, we investigated PL::A/P-DMS differences for the representation of latent choice values, a key driver of decision-making in the absence of task-relevant sensory information. We included trial-by-trial estimates of the difference between choice values (Δ*Q*), the sum of choice values (Σ*Q*), positive and negative reward prediction errors (+/−RPEs) in addition to the local reward rate (RR) over the last five trials to capture the strength of engagement in this self-initiated task. We found that the PL::P-DMS pathway more strongly encoded these internal value estimates (Fig. 7a, b), with the strongest drivers (Fig. S8a) being neurons modulated by difference in action values (Fig. 7c–f), action value sum (Fig. S8b) and those whose activity tracked with the local reward rate (RR; Fig. 7g–j). Interestingly, our encoding model mostly captured the slow shifting baseline of PL::P-DMS calcium activity that scaled with increasing Q-value difference or local reward rate (Fig. 7d–f, h–j) despite lacking clear event-related modulation (Fig. S8e, f). We found little evidence for activity modulation by RPE (Fig. S8c, d). Overall, these data imply that PL::P-DMS pathways more strongly represent temporally integrated internal measures of value than PL::A-DMS pathways.

Thus far, our data highlight a unique PL-striatal architecture defined by A-P striatal target that encodes complementary aspects of a value-based choice task. Our neural coding analysis makes several predictions about pathway-specific behavioral functions: 1. PL::A-DMS choice activity may shape current choice execution or instead provide an action-monitoring/expectation signal; 2. the persistent choice x positive outcome activity in PL::P-DMS could be used to drive positive reinforcement behavior; 3. PL::A-DMS negative outcome modulated neurons could be used to regulate choice strategies following negative outcome; 4. PL::P-DMS neurons encode temporally integrated signals for local reward rate and action value that may drive task engagement.

### PL::A-DMS pathways mediate future choice valuation, but not current choice selection or execution

**Fig. 8:**
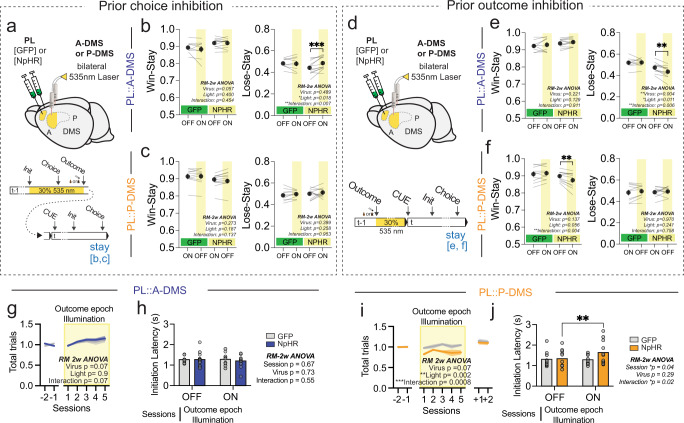
Optogenetic suppression during choice/outcome epoch impaired subsequent choice selection in a pathway-specific manner. **a** Schematic of surgery for pathway specific suppression and light delivery protocol for prior choice inhibition. **b** Changes in Win-stay (*left*) and Lose-stay (*right*) probabilities when light was ON versus OFF during choice on prior trials for PL::A-DMS circuits infected with either GFP or NpHR (black, mean ±SEM; light gray line, each animal). Yellow box indicates light delivered on prior choice epoch(GFP *n* = 9 animals, NpHR *n* = 12 animals, Šidák multiple comparison test). ***p < 0.001. **c** same as b, but for PL::P-DMS circuits (GFP *n* = 10 animals, NpHR *n* = 11 animals). **d** Schematic of surgery for pathway specific suppression and light delivery protocol for prior outcome inhibition. **e** Changes in Win-stay (*left*) and Lose-stay (*right*) probabilities when light was ON versus OFF after outcome on prior trials for PL::A-DMS circuits infected with either GFP or NpHR (black, mean ± SEM; light gray line, each animal). Yellow box indicates light delivered on prior outcome epoch (GFP *n* = 9 animals, NpHR *n* = 12 animals, Šidák multiple comparison test). **p < 0.01. **f** same as e, but PL::P-DMS circuits (GFP *n* = 10 animals, NpHR *n* = 11 animals, Šidák multiple comparison test). **g** Normalized number of total trials in sessions with and without outcome inhibition, where inhibition was delivered in the PL::A-DMS pathway for 30% of trials at random. Blue: NpHR; gray: GFP. of PL::A-DMS pathway in a random 30% of trials (yellow box). Solid line, mean; shaded area, ±SEM (GFP *n* = 9 animals, NpHR *n* = 12 animals). **h** Comparison of initiation latency (mean ±SEM) between sessions with (ON) or without (OFF) outcome epoch illumination of PL::A-DMS circuits from either GFP or NpHR group (GFP *n* = 9 animals, NpHR *n* = 12 animals). **i** same as g, but PL::P-DMS circuits (GFP *n* = 10 animals, NpHR *n* = 11 animals, Šidák multiple comparison test). **p < 0.01, ***p < 0.001. **j** same as **h**, but PL::P-DMS circuits (GFP *n*  = 10 animals, NpHR *n* = 11 animals, Šidák multiple comparison test). **p < 0.01.

To evaluate whether these divergent patterns of neural coding resulted in distinct functional contributions, we performed striatal subregion-specific optogenetic inhibition of PL terminals. We bilaterally injected PL cortex with AAV5-CamKII::NpHR3.0-EYFP, or AAV5-hSyn::EGFP for controls, and implanted 200 µm fiber optic cannulas bilaterally in either the A-DMS or P-DMS (Figs. 8a,dS9a–c). We designed two distinct light delivery protocols to assess the contribution of these PL-striatal circuits around choice and at outcome. We predicted that PL::A-DMS choice-associated activity might either have a role in the execution of current actions or instead provide an efference copy/anticipatory reward signal for the selected action that could be used to assess the resulting outcome and influence future action selection. We also predicted that manipulation of PL::P-DMS pathways would have no effects on choice selection, motor performance or evaluation, consistent with their lack of strong choice-associated modulation. To test these predictions, we activated NpHR from initiation through choice on a random 30% subset of trials (Fig. S10a). We found no evidence that optogenetic inhibition of either PL-DMS pathway throughout the choice period had any impact upon current trial value-based choice (Fig. S10b, d). To analyze effects on motor performance, we examined choice latency (the time from center port initiation until choice selection), observing no effect of optogenetic inhibition on choice latency distributions (Fig. S10c, e). To analyze the influence of choice-associated optogenetic suppression on subsequent action selection and performance, we relied on the strong influence of prior trial outcomes^33,34^, analyzing win-stay and lose-stay probabilities (Fig. 8b.c; see “Methods”). We found increased lose-stay behavior following choice activity suppression in prior trials for PL::A-DMS pathways (Fig. 8b) but no subsequent trial effects on motor performance (Fig. S10g). Consistent with our population coding data, optogenetic inhibition of PL terminals in P-DMS had no effect on either choice selection or execution for subsequent trials (Fig. 8c; Fig. S10h). Overall, these causal manipulations suggest that choice-epoch activity in PL::A-DMS is not related to action planning or execution, but instead provides an efference copy of actions or anticipatory signal for subsequent choice valuation.

### PL::DMS pathways divergently control response strategies to positive and negative outcomes

To directly evaluate the divergent functions of outcome-related PL::A/P-DMS activity, we optogenetically inhibited terminals in each striatal subregion during both positive and negative outcomes (Fig. 8d). While we did not observe any choice or performance changes from suppression of PL::A-DMS terminals following positive outcomes (Fig. 8e, Fig. S11b, d), we reliably observed a decrease in the win-stay probability from similar manipulations of the PL::P-DMS pathway (Fig. 8f). In contrast, we found that optogenetic suppression during negative outcomes of the PL::A-DMS, but not the PL::P-DMS, caused a decrease in lose-stay choice (Fig. 8e,f). This optogenetically-induced decrease in lose-stay behavior was consistently observed for PL::A-DMS inhibition across a range of reward probability environments (Fig. S11f). In low probability reward environments (*P*_rew_ = 0.4), inhibition of PL::A-DMS negative outcome signals led to less effective choice behavior following unrewarded trials while the opposite was true for high reward probability environments (*P*_rew_ = 1.0/0.85) (Fig. S11g). Finally, we also noted that PL::A-DMS inhibition disrupted the natural slowing of trial initiations observed following negative outcomes (Fig. S11c)^25,33,34^. These results support a model of divergent fronto-striatal control of outcome-based choice strategies, with PL::P-DMS activity mediating positive reinforcement and PL::A-DMS driving choice persistence in the face of negative outcomes.

### Temporally integrated PL::P-DMS neural activity supports task engagement

PL::P-DMS pathways were found to strongly encode action value differences and local reward rates, two temporally integrated measures of recent task outcomes. As the slow dynamics of these neural signals precluded precise optogenetic interrogation, we performed in-depth analysis with our second optogenetic paradigm, where inhibition was delivered for 6 s following outcomes (Fig. 8d). We assumed this manipulation would best reduce persistent activity and have broad effects on behavior, even outside of light trials. We measured the total number of completed trials as a proxy for task engagement, finding that outcome suppression of PL::P-DMS pathways on 30% of trials caused a decrease in the total number of completed trials for sessions where light was used (Fig. 8i). This effect was not observed in subsequent light-off sessions (Fig. 8i), during shorter choice suppression sessions (data not shown) and could not be explained by other typical motivational regulators such as body weight (Fig. S11j). Task disengagement was also manifest as increased initiation latencies in the PL::P-DMS outcome inhibition sessions (Fig. 8j) but not as overall slowing of motor performance (note unchanged choice latencies in Fig. S11k). In contrast, the PL::A-DMS pathways, which exhibited weaker internal value coding, did not impact task engagement as measured by total trials or initiation latencies (Fig. 8g, h, Fig. S11h, i). These results suggest that temporally integrated task value signals in PL::P-DMS pathways are important for driving global task engagement.

## Discussion

The dorsal striatum is a canonical set of circuits that interfaces much of the forebrain with downstream basal ganglia nuclei that select and modulate motor output^35^. Accordingly, neural processing within striatum is thought to be reflective of cortical activity^36^. Cortico-striatal projections are highly localized along the dorsal-ventral and medial-lateral axes^4^, but less so along the anterior-posterior striatal extent^3,11^. Here we sought to understand the implications of this architecture for cortico-striatal processing, focusing on prelimbic cortical connections to dorsomedial striatum. As for most DMS-targeting afferent populations, we found that PL cortex formed non-overlapping circuits according to A-P target. Prelimbic to A/P-DMS could be separated based on their local synaptic connectivity to striatal SPN subtypes as well as biases in their afferent drivers. In vivo imaging demonstrated that these two populations divided encoding of key behavioral variables for goal-directed choice. PL::A-DMS pathways strongly encoded selected choices and negative outcomes, while PL::P-DMS pathways strongly encoded internal value representations and an integrated positive outcome/choice signal. Target- and temporally- specific optogenetic manipulations further confirmed the functional divergence of these fronto-striatal circuits, with PL::A-DMS pathways providing choice and negative outcome monitoring while PL::P-DMS pathways supporting task engagement and reinforcement by positive outcomes.

In our attempts to understand potential functional differences of PL::A/P-DMS pathways, we first investigated whether these circuits had distinct synaptic connectivity to their local striatal microcircuits. We found that evoked monosynaptic excitatory currents onto dSPNs, regardless of DMS compartment, were larger than those onto iSPNs (Fig. 2b). While these data are consistent with dSPN bias of prefrontal populations found in original rabies virus mediated striatal tracing studies^37^, and surprising considering more targeted DMS tracings^38^, all transsynaptic tracing studies remain limited in their ability to predict synaptic efficacy^38^. Despite both A/P-DMS dSPNs receiving strong excitation from their respective PL drivers, only PL projections to A-DMS strongly recruited feed-forward inhibition onto dSPNs (Fig. 2c). These data may help explain the curious finding that prefrontal input suppression was associated with increases in SPN in vivo firing rates^39^. Together these data suggest that while PL::P-DMS circuits largely rely on dSPN excitation, PL::A-DMS circuits can recruit both SPN subtypes and may further regulate dSPN activity depending on the status of local striatal inhibitory neurons. Subtype-specific SPN optogenetic manipulations have demonstrated that A-DMS dSPNs exert robust, bi-directional control of flexible responding in contrast to iSPNs in a reversal task, highlighting the potential importance of a circuit arrangement that could bidirectionally toggle dSPN activity^13^. Further work combining PL::A-DMS terminal inhibition with striatal SPN recordings should clarify the importance of this feedforward microcircuit as well as the effects of our PL optogenetic manipulation on striatal circuit output.

Feedback-driven value-based behaviors require specific response strategies to positive and negative outcomes, estimation and retention of value estimates for actions, the appropriate assignment of credit for temporally displaced choice and outcome, as well as regulation of motivation, performance, and task engagement. Here we provide evidence that prefrontal connections to the DMS supports many of these evaluative functions and do so in a distributed manner across A-P striatal targets.

Our Ca^2+^ imaging data demonstrated that PL populations projecting to A-DMS contain neurons tuned to multiple sensorimotor components of our operant task. While start cue and subsequent initiation approach were represented by small populations, we found that a substantial number of PL::A-DMS neurons were modulated around choice selection (Fig. 5c). This activity was not related to encoding head velocity or the specific turning motions required to execute choice. Averaged choice-associated kernels revealed larger contralateral than ipsilateral choice signals that began just before choice was registered (Fig. S6b). These data are consistent with previous work showing only weak neural signals for upcoming choice in medial prefrontal cortex (mPFC), suggesting activity in this region doesn’t significantly contribute to action planning in trial and error tasks^40^. If not directly encoding a decision variable for choice or ongoing motor output, this signal may instead represent an evaluative process - either an efference copy of the selected action or an expectation of upcoming outcome. Striatal-targeting efference signals have been proposed to function together with cortical representations of the environment to bind context, selected action and outcome^1,41^.

We directly tested the functional importance of choice-associated modulation via optogenetic inhibition of PL terminals within the A-DMS, finding that while bilateral optogenetic disruption of these circuits around the choice period had no effect on current trial choice or motor performance, this manipulation specifically altered choices on trials following negative outcomes (Fig. 8b). These data suggest PL::A-DMS signals at choice provide either an efference copy of selected actions or an anticipatory signal of outcome that is utilized to update choice values on subsequent trials. Interestingly, our choice-associated signals only seemed relevant following negative outcomes, as manipulations did not alter win-stay probabilities (Fig. 8b). These data are consistent with the biased responding of PL::A-DMS pathways towards negative outcomes (see below), suggesting common valence processing in this pathway. Recently, PL neurons that project to the nucleus accumbens core were shown to exhibit choice modulation that progressed sequentially through the population, bridging choice and outcome periods^42^. In contrast to our results, optogenetic activation of PL-NAc throughout the trial altered subsequent responses following both positive and negative outcomes.

Outcome monitoring is thought to be a crucial function of prefrontal cortical circuitry, influencing how animals use subsequent sensory information^43,44^ and select future actions^25,34,45^. While the PL cortex has been suggested to provide both positive and negative feedback signals to shape behavior^46^, our experiments reveal a distribution of these functions according to DMS target, with positive outcomes encoded by both pathways and negative outcome encoding mostly by PL::A-DMS. Both PL-DMS pathways exhibited encoding of brief (~1 s) neuronal responses to positive outcomes (Fig. S7a–h), while PL::P-DMS more strongly encoded positive outcomes that followed specific choices (Ch x O+ interaction; Fig. 6f). Interestingly, activity patterns for Ch x O+ coding exhibited unique temporal patterns according to PL circuit, with a persistent (>5 s on average) activity in PL::P-DMS neurons (Fig. 6g). We hypothesized that this activity would be central to positive reinforcement behavior, either via providing an eligibility trace for plasticity or by directly influencing ensuing decision processes. To test this, we optogenetically inhibited PL::P-DMS continuously for 6 s. following trial outcome, observing that stay-behavior was reduced following positive outcomes with no change in choice for manipulation following negative outcomes. These data are strongly consistent with seminal experiments showing the P-DMS to be central to outcome-driven action selection^8,9^. Furthermore, it seems possible that this prolonged Ch x O+ activity may explain the value-based learning deficits observed upon chronic chemogenetic-mediated suppression of PL-P::DMS pathways^6^.

Another surprising result of our work was the biased representation of negative outcomes by PL::A-DMS pathways. Averaged negative outcome kernels in this population displayed a delayed onset and persistent activity lasting over 5 s, consistent with an outcome feedback signal as opposed to reward port approach (Fig. 6l). While there are numerous examples of outcome encoding in rodent PL cortex for negative valence, most cases involved aversive stimuli such as foot-shocks or air puffs^47^. A gambling task in rats, where risky maze arms had lower probability/higher reward outcomes, elicited prolonged bouts of firing in PL neurons at negative outcome that supported risky choice^48^. Choice monitoring activity was also seen at outcome in PL neurons which supported cognitive flexibility during set-shifting tasks^44^. We found that specific optogenetic inhibition of negative outcome signals in PL::A-DMS pathways reliably decreased stay behaviors following a prior loss (i.e., increased choice switching), while having no choice effects following prior positive outcomes (Fig. 8b). The ability of PL::A-DMS outcome activity to support choice persistence following losses was independent of reward context, as optogenetic inhibition always decreased lose-stay behavior over a range of session reward probabilities (Fig. S11f). Thus, this optogenetic manipulation improved overall performance in high reward probability environments, but impaired it in lower reward probability scenarios, where lose-stay behavior is adaptive (Fig. S11g). This data argues against a flexible, context-dependent role for PL::A-DMS circuits in mediating behavioral strategies following negative outcome. Furthermore, these functional effects contrast with negative outcome-tuned neurons in the ACC, which have been shown to implement choice switching in many species^49,50^. While response persistence in the face of negative outcomes is essential in sparse reward environments (see Fig. S11g Prew = 0.4), left unchecked this tendency could clearly impair value-based function. This raises the question of whether mouse models of neuropsychiatric disease characterized by perseverative choice abnormalities exhibit dysregulation of PL::A-DMS pathways.

Internal representation of choice value and local reward availability are key determinants of behavior in dynamic foraging tasks^25,34^. Our results suggest that PL::P-DMS pathways more strongly encode these behavioral parameters as compared with PL::A-DMS pathways. We found that relative value signals tracked strongly with the baseline, but not phasic components of cellular calcium signals (Fig. 7d, h, Fig. S8e, f). This ΔQ-encoding PL population may represent a previously identified PL-DMS population that stably represented relative value via persistent baseline spiking activity^25^. While we also identified neural signals encoding total choice value (ΣQ) as in Bari et al., our inability to control trial initiation precluded investigation into the relative persistence of these distinct value signals^25^. Engagement in self-initiated foraging tasks is strongly modulated by local reward environment, a variable we captured with a local average of the reward rate. Again, we found that PL::P-DMS pathways more strongly encoded this feature as compared to PL::A-DMS pathways. The persistent nature of value coding in these pathways made phasic optogenetic manipulation difficult. To circumvent this, we looked at all trials in sessions where inhibition was delivered in 30% of trials for 6 s. after outcome, reasoning that prolonged inhibition should sufficiently alter persistent neural signals to impact the overall behavior (including non-light trials) in the session. Indeed, we found that post-outcome inhibition was able to both reduce the total number of initiated trials and reduce the win-stay probability in non-light trials, suggesting the involvement of reward-rate and ΔQ-encoding PL::P-DMS populations, respectively. It is interesting to hypothesize that the reduction in task engagement caused by disruption of this pathway may share a common cause with the reduced responding seen in earlier P-DMS lesion studies^8^.

One challenge in comparing our data to existing work on DMS function along the anterior-posterior axis is these prior study’s satiety-based devaluation methods to examine changes in action-value associations^8,9,12^. While our initial Kir2.1 manipulations within DMS confirmed the importance of P-DMS for devaluation-based decreases in responding (Fig. S3g–j), most of our study uses sequential, performance-driven reversal of rewarded choice. We opted for this task to increase the number of trials in which to image PL circuit activity as choice value fluctuated. While we have tried whenever possible to drawn potential parallels between lesion and DREADDs studies of A/P-DMS, further work is necessary to know whether identical neural mechanisms are employed between satiety-based devaluation and the serial shifting of valued choices used here. Furthermore, care should be taken in making comparisons between direct striatal manipulations (Fig. S3) and the projection terminal optogenetic manipulations made here (Fig. 8), as these perturb only one of many signals integrated by a given striatal region. We also cannot entirely rule out that our optogenetic manipulations may include a portion of ventral ACC in addition to PL, although we believe these effects are minimal. Finally, we should note that despite the strong superficial layer bias of both PL-DMS pathways, we cannot ascribe layer-specific functional effects on behavior with our approaches. Further work will be necessary to appreciate how superficial and deep PL cortical neurons contribute to the observed functional diversification.

Our work adds to recent studies demonstrating a range of behavioral functions for PL cortical microcircuits defined by target area^21–23,42,44^. Nevertheless, the mechanisms underlying the functional diversification of these circuits remain unclear, with potential candidates including differences in molecular composition, local cortical network connectivity or long-range afferent drivers. While evidence exists for target-specific transcriptional differences in PL cortex^21^, other analyses have shown diverse PL functions emerging from molecularly homogenous populations^23^. Circuit-specific transcriptional profiling could reveal whether molecular diversity can account for divergent PL-DMS pathway activity. Differences in afferent connectivity may result from circuit-specific differences in local inhibitory control^51^ or long-range excitatory projections. We used 2-stage retrograde tracing to map afferent populations that synapsed on PL neurons defined by A/P-DMS target (Fig. 2d–f), finding that ACCv, RSP cortex, and OFC were strongly biased in connecting to PL::A-DMS populations while M2 favored PL::P-DMS neurons. Upstream manipulations will be necessary to test whether prolonged choice^45^ or value^52^ encoding in M2 supports persistent Ch x O+ signaling in PL::P-DMS neurons, while enhanced ACCv, RSP, and OFC connectivity to PL::A-DMS supports negative outcome associated activity. Similar tracing approaches have highlighted the importance of ACCv connectivity to deep PL layers projecting to NAc for outcome monitoring during cognitive flexibility tasks^44^.

In this study, we revealed distinct functional roles of two PL::DMS circuits in value-based decision making (Fig. S12). Our initial tracing data showed a surprising number of cortical and thalamic regions have distinct, yet intermingled populations projecting to A/P-DMS (Fig. S1). Furthermore, our results suggest that despite the bias towards a greater density of PL fibers in the A-DMS, manipulations of PL-pDMS circuits can have distinct, but similarly penetrant behavioral effects. Future work should explore potential computational advantages afforded by this arrangement. It is presently unclear whether anterior and posterior striatal subregions might work coordinately or antagonistically to control behavior, which would be an important starting point for our understanding. Either way, this organization could permit appropriate and flexible coordination of A/P-DMS targeting populations via local-circuit interactions in cortex or thalamus. Alternatively, these parallel processing paths may be integrated via downstream basal ganglia components.

## Methods

### Animal

Animal experiment procedures were approved by the *University of Pennsylvania Institutional Animal Care and Use Committee*, and all experiments were conducted in accordance with the *National Institutes of Health Guidelines for the Use of Animals*. Unless otherwise noted, animals (C57BL/6NCrl from Charles River laboratory, strain code 027; Adora-Cre mice from Jackson Laboratory, B6.FVB(Cg)-Tg(Adora2a-cre)KG139Gsat/Mmcd) were grouped with littermates on a 12:12 light-dark cycle and provided *ad libitum* food and water. Given the potential for impacts of the estrous cycle on goal-directed behavior, all experiments were conducted on naive male mice.

### Stereotaxic surgery

Intracranial surgery was conducted on a stereotaxic surgery frame (Kopf Instrument, Model 1900) under isoflurane anesthesia (1.5–2% + oxygen 1 L/min). Animal body temperature was maintained at 30 °C during surgery using a feedback thermocontroller (Harvard apparatus, #50722 F). Skin was cleaned with Nair hair remover followed by application of betadine to disinfect the area. Prior to surgery, 2 mg/kg bupivacaine was administered subcutaneously, and the mouse was given a single dose of meloxicam (5 mg/kg). Skin was carefully opened along the anterior-posterior midline, bregma was set to zero based on skull balance. A craniotomy was performed with a drill above the target site. Virus or Tracer was loaded into mineral oil (Sigma-Aldrich, M3516)-filled glass pipette (WPI, TW100F-3) and delivered at rate 30 nl/min using a micro-infusion pump (Harvard Apparatus, #70-3007). At least 5 minutes after infusion, the pipette was slowly withdrawn (1 mm/min) from the brain, and the skin was sutured. Animals were monitored up to 1 h following regaining of consciousness, then transferred to the home cage and monitored after 24 h, 48 h and 72 h. Injection coordinates, A-DMS: AP + 1.2 mm, ML + 1.35 mm, DV −2.7 mm; P-DMS: AP −0.3 mm, ML + 1.95 mm, DV −2.2 mm; PL: AP + 2.0 mm, ML + 0.35 mm, DV −1.7 mm

### Anatomical Tracing

For mapping PL synaptic terminals in DMS (anterograde tracing), a 1:1 mixture of AAV5-CaMKii::Cre (Penn Vector Core, CS1185L, 1.3e13GC/ml) + AAVdj-EF1a::Flex-Synaptophysin-mRuby (in-house production, non-titered) viruses was injected into PL. For mapping retrogradely labeled neurons, a mixture of AAV1-Syn::Cre (Penn Vector Core, CS1352, 3.3e13GC/ml) + AAVdj-EF1a::DIO-RVG + AAVdj-EF1a::Flex-TVA-mCherry (both in-house productions, non-titered) was injected into A-DMS and Alexa647-conjugated Cholera toxin subunit-B (Invitrogen C34778, 75 nL, 1ug/μL) was injected into P-DMS. Seven days later, EnvA G-Deleted Rabies-eGFP (Salk institute virus core, 32635, 5.0e7TU/mL) was injected into A-DMS. This trans-synaptic tracing approach allowed us to avoid retrogradely labeling neurons via fiber-of-passage uptake (cholera toxin-based tracers undergo passive membrane incorporation) in the A-DMS, where P-DMS fibers would be traversing^53^. For CTB only tracing (Fig. S1), CTB488 (Invitrogen C34775, 75 nL, 1ug/μL) and CTB555 (Invitrogen C34776, 75 nL, 1 μg/μL) were injected into A-DMS and P-DMS respectively. For mapping 2nd-tier projections to PL::DMS pathway, retroAAV2-hSyn::3xFlag-Cre (in-house production, non-titered) was injected into the A- or P-DMS followed by a mixture of AAVdj-EF1a::DIO-RVG + AAVdj-EF1a::Flex-TVA-mCherry into PL. Seven days later, EnvA G-Deleted Rabies-eGFP was injected into PL.

After viral injection, 7 days (for Rabies virus) or 3 weeks (for AAV) were allowed for viral expression, animals were deeply anaesthetized with i.p injection of 100 μL pentobarbital sodium (Nembutal, 50 mg/mL) and transcardially perfused with PBS followed by formalin (10%). Brains were removed and post-fixed in formalin (Thermo Fisher Scientific, SF1004) overnight, then transferred to PBS. Brains were sectioned coronally at 50 µm then brain slices were mounted on slide glasses and covered with fluoromount solution (SouthernBiotech, #0100-01) for imaging.

Stitched large-field images were obtained with a 4× objective (Olympus, 4×, 0.16NA) on an epi-fluorescent microscope (Olympus, BX63). Fluorescence-positive neurons were counted using automated object detection (NeuroInfo Suite, v2021.). Three-dimensional brain images were then reconstructed using NeuroInfo software (MBF bioscience), which registered individual slices to the Allen Institute reference brain atlas (Allen mouse common coordinate framework; CCFv3)^54^.

### Immunohistochemistry

At room temperature, free floating brain slices were permeabilized in 0.6% Triton x-100 and blocked with 6% normal goat serum (Jackson ImmunoResearch, 005-000-121) in PBS for 1 h. Samples were incubated in primary antibody solution (Rat anti-CTIP2, 1:500, Abcam, ab18465) overnight in 0.2% Triton x-100 and 2% NGS in PBS. Slices were washed then incubated in secondary antibody solution (Goat anti-rat IgG-alexa555 conjugated, 1:500, Invitrogen, A48263) for 1 h in 0.2% Triton x-100 and 2% NGS in PBS, then mounted and imaged.

### Electrophysiology

For slice physiology, channelrhodopsin-expressing virus (AAV.DJ-hSyn-ChiEF-2a-Venus) was injected in PL. To visualize specific striatal cell types, AAVdj-EF1a::DO/DIO-GFP/tdTomato was injected into the A/P-DMS in Adora2A-Cre mice (KG139Gsat). All mice were allowed to recover for 4-5 weeks prior to recording. Mice were deeply anesthetized and trans-cardially perfused with ice-cold aCSF containing (in mM): 124 NaCl, 2.5 KCl, 1.2 HaH_2_PO_4_, 24 NaHCO_3_, 5 HEPES, 13 Glucose, 1.3 MgSO_4_, 2.5 CaCl_2_. After perfusion, the brain was quickly removed, submerged, and coronally sectioned on a vibratome (VT1200s, Leica) at 250 μm thickness in ice-cold aCSF. Slices were transferred to NMDG based recovery solution at 32 °C of the following composition (in mM): 92 NMDG, 2.5 KCl, 1.2 NaH_2_PO_4_, 30 NaHCO_3_, 20 HEPES, 25 Glucose, 5 Sodium ascorbate, 2 Thiourea, 3 Sodium pyruvate, 10 MgSO_4_, 0.5 CaCl_2_. After 12–15 min recovery, slices were transferred to room temperature aCSF chamber (20–22 °C) and left for at least 1 h before recording. Following recovery, slices were placed in a recording chamber, fully submerged at a flow rate of 1.4–1.6 mL/min, and maintained at 29–30 °C in oxygenated (95% O_2_, 5% CO_2_) aCSF containing AP5 (50 µM, Tocris).

For voltage-clamp recordings, recording pipettes were pulled from borosilicate glass (World Precision Instruments, TW150-3) that had a tip resistance of 3~5 MΩ when filled with internal solution containing (in mM) 130 CsMeSO4, 5 CsCl, 10 HEPES, 2.5 MgCl, 0.6 EGTA, 1 QX-314, 10 Na-Phosphocreatine, 4 NaATP, and 0.3 NaGTP 0.1 spermine (pH adjusted to 7.3–7.4 using CsOH). Striatal neurons were identified under visual control using IR-DIC optics (Olympus, BX51). Visual identification of D1-MSN/D2-MSN was based on expression of GFP/tdTomato (Chroma, #49002, #49005). Cells were voltage clamped at either −56 mV(Cl^-^ reversal potential) and at +10 mV (cation reversal potential), which were determined in prior experiments. For current-clamp recordings, recording pipette filled with internal solution containing (in mM) 140 K-gluconate, 5KCl, 0.2 EGTA, 2 MgCl_2_, 10 HEPES, 4 MgATP, 0.3 NaGTP, 10 2Na-Phosphocreatine (pH adjusted to 7.3-7.4 using KOH). Recordings were performed using a MultiClamp 700B (Molecular Devices) and Igor7 (WaveMetrics; recording artist addon, developed by Richard C Gerkin, github: https://github.com/rgerkin/recording-artist), filtered at 2.8 kHz and digitized at 10 kHz. ChIEF expressing axon terminals were stimulated with brief (5 ms) pulses of 473 nm blue light from a collimated LED illuminator (Thorlabs, LED4D067, DC4100). Input and series resistance were monitored continuously, and experiments were discarded if either parameter changed by >20%. NBQX(10 µM, Tocris) PTX (100 µM, Sigma-Aldrich) were applied if necessary.

### Behavioral equipment

Behavior training was conducted utilizing a custom built 3-port operant chamber (dimensions 7.5 L × 5.5 W × 5.13 H inches, Sanworks LLC, NY). Each port is controlled by a TTL signal from the state machine consisting of white LED light, infrared beam break detector and liquid outlet. The center port was designated as a reward delivery outlet using a pinch valve (225P011-21, NResearch, NJ). All behavior chambers were enclosed in sound-attenuating boxes (PSIB27, Pyle, NY). Behavior protocols were controlled by Bpod software (https://github.com/sanworks/Bpod) in MATLAB (MathWorks). All port entries and events were recorded by the Bpod State Machine during behavioral sessions.

### Behavioral training

To increase operant responding, total calorie consumption was reduced over 1 week to reach 85–90% body weight, a level maintained throughout operant training. Animals were habituated to behavior chambers for at least 2-days prior to training. Each day, animals were given 45 min of exposure to the behavioral box with chocolate milk (Boost Original ready to drink, rich chocolate nutritional shake, Nestle) delivery from the center port spaced 20 s. apart. Following the habituation period, animals performed light-guided sessions as follows: (1) center port light indicated the beginning of a trial; (2) trial initiation via a center poke led to illumination of a randomly selected side port; (3) appropriate selection of the lit port within 3 s led to illumination of the center port and delivery of 12 μl of Boost at this location; (4) selection of the unlit alternative led to illumination of the center port without concomitant dispensing of reward. Each trial was separated by a 5 s. ITI in which all chamber lights were extinguished. Sessions lasted 1 h with no trial limits. Animals were considered to reach criteria with >200 completed trials per session.

### Two-alternative forced choice task

After reaching criteria performance levels in light-guided training, mice progressed to a 2-alternative forced choice task structured as follows: (1) center port light indicated the beginning of a trial; (2) a 500 ms. holding period (sequentially increased from 0, 100, and 300 ms) in the center port triggered the illumination of both side ports; (3) animals had a 3 s. window to register either left or right port choice. When animals failed to make a choice in this period this resulted in an omission, which was followed by a 3 s timeout and required the animal to reinitiate the trial. (4) successful registration of a choice was followed by 0.5 s delay period ending in the outcome period (*P*_outcome_ = 85%). Correct choice resulted in delivering 12 μL Boost from the center port with 85% chance while incorrect choice resulted a in 500 ms punishment tone (white noise) with 3 s timeouts, also with 85% chance. In the remaining 15% of trials, animals didn’t receive any outcome (reward or punishment). Each trial was separated by a 3 s. ITI in which all chamber lights were extinguished. To prevent outcome-insensitive behavior, past-reward history was monitored in a 10-trial moving window, and rewarded side was switched when 8 of the last 10 choices were to the currently rewarded port. Sessions lasted for either 45 min. (1-p imaging) or 1 h (optogenetic manipulations). We utilized a relative reward-stay value >2 to decide when to move mice on to recording sessions. Relative reward stay was defined as:

1 $${Relative\; reward\; stay}={{{{\mathrm{ln}}}}}\left(\frac{\frac{P\left({WinStay}\right)}{1-P\left({WinStay}\right)}}{\frac{P\left({LoseStay}\right)}{1-P\left({LoseStay}\right)}}\right)$$

### Behavioral experiments with Kir2.1

For region specific silencing experiment, we overexpressed Kir2.1 using mixture of AAVdj-EF1a::DIO-Kir2.1-zsGreen (in-house production, non-titered) and AAV5-CaMKii::Cre. To measure an ability to acquire instrumental learning, following the habituation period, animals performed instrumental acquisition sessions as follows: (1) center port light indicated the beginning of a trial; (2) trial initiation via a center poke led to illumination of a both side port; (3) selection to pre-determined port within 3 s led to illumination of the center port and delivery of 12ul of Boost at this location. pre-determined port did not change through a session; (4) selection of another side port led to illumination of the center port without concomitant dispensing of reward. Each trial was separated by a 5 s. ITI in which all chamber lights were extinguished. Sessions lasted 1 h with no trial limits. To compare response rate between pre-fed with substance for reward (devalue; DV) and pre-fed with food chow (value; V), mice that were trained with fixed-ratio 20 schedule for 2–3 days were exposed to each substance for 1 h in different training day in home cage followed by behavior test under extinction protocol without outcome for 15 min.

### Modeling of animal choice

We adapted a simple Q-learning Reinforcement Learning Model with three parameters to fit the behavioral data produced by the serial reversal task. Mouse choice and outcome history were the primary inputs of the model. The values of the choice alternatives were initiated at 0 and updated as follows. For the chosen port,

2 $${Q}_{t+1}={Q}_{t}+\alpha ({R}_{t}-{Q}_{t})$$

while for the non-chosen port the *Q*-value decayed towards zero, implementing a kind of “forgetting”,

3 $${{{{{{\rm{Q}}}}}}}_{{{{{{\rm{t}}}}}}+1}={{{{{{\rm{Q}}}}}}}_{{{{{{\rm{t}}}}}}}(1-\varphi )$$

where *Q*_t_ is the value of the action taken on trial t of each choice and R is the actual reward received in trial t. The parameter α thus constituted a learning rate, while the parameter φ constituted a forgetting rate for the non-chosen port. The decision process mapping the Q-values to the probability of choosing one port over the other was modeled with a softmax rule:

4 $${P}_{A}\left(t\right)=\frac{1}{1+\exp \left[-\beta \left({Q}_{A}\left(t\right)-{Q}_{B}\left(t\right)\right)\right]}$$

Here *β* is an inverse temperature parameter controlling the degree to which choices are biased by estimated value. High values for *β* indicate that mice more readily exploit differences in action values between the alternatives, while lower values suggest that mice exhibit more exploratory behavior. To fit this model to our choice data, we used the *fmincon* function in MATLAB to minimize the negative log likelihood of models using our parameters (α, β, φ).). As a special case of this RL model, we obtained a simpler version without forgetting by imposing φ = 0.

As a further check on the performance of the RL model, we compared its goodness of fit and predictive power with that of three other models (Figs. 3c, S4d). The first was a logistic regression model (LogReg) that predicted the upcoming choice based on the choices and outcomes of the previous 5 trials. More formally, the logistic regression model assigned the following probability to choosing the port on the right:

5 $$P\left(t\right)=\frac{1}{1+{e}^{-h\left(t\right)}}$$

where

6 $$h\left(t\right)={\beta }_{0}+\mathop{\sum }\limits_{i=1}^{5}{\beta }_{1,i}c\left(t-i\right)+\mathop{\sum }\limits_{i=1}^{5}{\beta }_{2,i}r\left(t-i\right)$$

with *c(t)* being the mouse choice on trial *t* (*c* = 1 for right choice, *c* = −1 for left choice), and *r(t)* representing the rewarded side (*r(t)* *=* 1 if trial *t* lead to a reward following a right choice, *r(t)* *=* −1 if it leads to a reward following a left choice, and *r(t)=0* if there was no reward). Because of the symmetrical encoding of left and right choice, the regression coefficient *β*_*1,i*_ captures the tendency of the mouse to repeat the choice operated at trial *t-i*, and *β*_*2,i*_ captures the tendency to choose the side that was rewarded at trial *t-i*.

The second model we considered for this comparison was another logistic regression (rLogReg), where the weights on past rewards were constrained to decay exponentially in time:^29^

7 $$h\left(t+1\right)=\gamma+\alpha c\left(t\right)+\beta \mathop{\sum }\limits_{i=0}^{{{\infty }}}{e}^{-i/\tau }r\left(t-i\right)$$

This model has four parameters (α, β, γ, τ). α represents a tendency to repeat the previous trial’s choice (“choice stickiness”); β is a general sensitivity to the history of reward; τ controls how far back in time the memory of past rewards extends; and γ is a general bias towards one choice or the other. This model is called “recursive logistic regression” because it allows for a recursive definition of a “state estimate” ρ:

8 $$\rho \left(t\right)=\beta \mathop{\sum }\limits_{i=0}^{{{\infty }}}{e}^{-i/\tau }r\left(t-i\right)=\beta r\left(t\right)+{e}^{-1/\tau }\rho \left(t-1\right)$$

The third model we considered in the comparison with the RL model was a simple win-stay, lose-shift strategy (WS-LSh), made stochastic by the presence of a lapse rate ε. Under this model, on any trial the mouse performed a random choice with probability ε. If this was not the case (with probability 1−ε), the mouse repeated the choice made on the previous trial if that choice had been rewarded (win-stay) and made the opposite choice otherwise (lose-shift).

We compared these three models with the Q-learning model by computing the Akaike Information Criterion (AIC), which adjusts the deviance of a model to penalize complex models with a larger number of parameters. The recursive logistic regression model had comparable AIC to that of the RL model, while the full logistic regression and the win-stay/lose-shift model both performed worse (Fig. 3c). Comparison with the Bayesian Information Criterion (BIC) yielded similar results (Fig. S4d).

### Tracking animal body parts

To track the animals’ body movement, behavioral sessions were recorded using an overhead webcam (Brio, Logitech). Recording was performed using built-in software in miniscope software, frame rate was fixed at 30 fps/s. To extract head location, we averaged coordinates from right and left ear that were detected by DeepLabCut (DLC) software (Version 2.2.0.6)^32^. We labeled 8 reference videos and more than 50 frames/video to train the DLC network using *resnet_50 network* (built-in, default augmentation method). Head velocity was estimated at any point in time as the difference between head position in two successive frames, multiplied by the frame rate. In order to use head velocity as a predictor in the encoding model, the resulting time series was linearly interpolated to allow for synchronization with the miniscope imaging timestamps.

### 1-p Imaging

To record calcium signals from PL neurons targeting specific striatal subregions, retroAAV2/EF1a-3xFlag-Cre^55^ was unilaterally injected to A- or P-DMS, together with AAV1/hSyn-Flex::GCaMP7f-WPRE(Addgene,104492-AAV1, 1.0e13vg/ml)^56^ injection into PL. Prior to relay GRIN lens (1 mm × 4 mm, Inscopix, 1050-002176) implantation in PL, upper prefrontal tissue was gently aspirated using a glass pipette until reaching 0.5 mm above target site. Following tissue aspiration, the GRIN lens was slowly lowered (100 μm/min) until 0.3 mm above from the target site. Dental cement (Geristore™) was used to create a foundation around the GRIN lens, and the remaining exposed GRIN lens was covered with silicone paste to prevent scratches. After surgery, animals were transferred to a single housed cage, where their status was monitored until movement recovery. The anti-inflammatory Meloxicam (5 mg/kg) was applied subcutaneously daily for >1 week, and animals were carefully monitored. Four to six weeks following GRIN lens implantation, the miniscope baseplate was installed under 1-p imaging (UCLA miniscope v3.0)^57^ to locate fields of view (FOV) with robust GCaMP7f expression. Once a FOV was selected, the baseplate was fixed with dental cement to make a crown. Baseplates were covered with a cap, and animals were subsequently returned to the home cage.

### Signal processing

To extract calcium traces from imaging videos, we utilized MIN1PIPE (v2 alpha, https://github.com/JinghaoLu/MIN1PIPE/tree/v2-alpha) for motion correction (Hierarchical non-rigid movement correction), segmentation (GMM, LSTM classifier), and signal deconvolution (CNMF identifier)^31^. Each ROI selected by MIN1PIPE was individually reviewed to ensure somatic morphology and remove repeated selection of the same neuron’s proximal dendrites.

### Neural encoding model

To analyze task-relevant neural activity we designed a neural encoding model based on a linear combination of event-based and continuous predictors^58,59^. Here, by “event-based” we mean a predictor that is associated to a particular event (e.g., reward delivery), and is characterized in the fitted model by a temporal kernel defined over a fixed time window around the event; therefore, this type of predictors will contribute to the predicted activity only in proximity of the associated event. By “continuous” we mean a predictor that simply takes on a particular value at any given point in time, such as the head velocity; therefore, this type of predictors will generally contribute to the predicted activity at any point in time (note that some of our continuous predictors are not continuous in a mathematical sense, because they change in a stepwise manner—see below).

More precisely, we modeled the neural activity of a given neuron as:

9 $$y(t)=\alpha+\sum {\beta }_{i}{x}_{i}(t)+{\sum }^{}{\sum }^{}\sum {\gamma }_{k}^{(j)}{f}_{k}^{(j)}(t-{t}_{n}^{(j)})+{\epsilon }$$

where *α*, *β*, and *γ* are parameters to be fitted, the index *i* runs over all continuous predictors, and ${x}_{i}\left(t\right)$ is the time course of the *i*th continuous predictor. *T*he index *j* runs over all event-based predictors, ${f}_{k}^{\left(j\right)}$ is the *k*th function of the temporal basis set associated with the *j*th event-based predictor, and ${t}_{n}^{\left(j\right)}$ is the time of occurrence of the *n*th event of the *j*th type. ε represents some normally-distributed noise. As temporal basis functions  ${f}_{k}^{\left(j\right)}$ we used cubic b-splines, characterized by time windows and number of knots that depended on the predictor (see Supplementary Table 1). The model estimates the average calcium activity as a linear superposition of transient behavioral contributions (either kernels or persistent effects) from distinct predictors, thus discounting overlapping transients from other event types in the estimate of each individual kernel. This approach takes advantage of the trial-by-trial variability in the timing of individual events in assigning signals that on some trials exhibit substantial overlap.

We included episodic directly measurable variables, including trial start cue, self-initiation, choice, and outcome, which were represented by spline-based, temporally expanded kernels (Fig. 4c). As a proxy for the animal’s body movements, we included head velocity as a continuous predictor, which at any point in time took on a value corresponding to the instantaneous velocity estimate extracted from overhead body imaging (see above, “tracking animal body parts”). In addition, we sought to identify neural signals encoding relevant internal value information likely guiding choice^58^. To do this we fit our choice data with a Q-learning reinforcement model with forgetting (see above, “Reinforcement learning model”) and used *Q* values and reward prediction errors as internal behavioral variables. Specifically, we included trial-by-trial ΣQ (*Q*_left_ + *Q*_right_) and Δ*Q* (*Q*_left_ − *Q*_right_) values (as continuous predictors that stayed constant throughout the trial and changed their value instantaneously at outcome, consistent with the RL model) and reward prediction errors (kernels tethered to the outcome). Finally, we included a local reward rate averaged over the prior 5 trials as a continuous behavioral variable (in units of μl/min). We fit these regression parameters using a generalized linear model with near-lasso regularization (elastic net, alpha = 0.95) to achieve sparse regression weights, using the *glmnet* library^59^ (wrapped for MATLAB usage with custom software available at 10.5281/zenodo.3568314). Details on the representation of each predictor in the design matrix of the model are given in Supplementary Table 1. For each fitted trace, the shrinkage hyperparameter λ controlling the strength of the elastic net regularization was selected by 50-fold cross-validation. Following established practice (Hastie, 2008), given the maximum value of the (cross-validated) fraction of variance explained (FVE) over possible values of λ and its standard deviation across folds, we selected the largest value of λ such that its associated FVE was larger than the maximum minus one standard deviation, thus selecting the “simplest” model in the neighborhood of the best-fitting one. The cross-validation folds were stratified by experimental trial, so that each trial was represented roughly equally in each fold, and the data was grouped in 200 ms-long temporal chunks (typically corresponding to about 4 imaging frames) for the purpose of cross-validation, to reduce the number of temporally adjacent data samples in different cross-validation folds^60^.

We fitted an independent model to each recorded neuron. Using the model, the fraction of variance explained (FVE) was calculated by comparing the full model and actual calcium signal trace as an indicator of the accuracy of model prediction. To exclude non-task relevant neurons, we limited further analyses to those with at least 5% FVE. To assess contribution from a certain predictor, we calculated a tuning index defined as FVE(Full) – FVE (reduced), where FVE (reduced) is the FVE of the reduced model obtained by removing the predictor of interest from the full model (Fig. S5a). Tuning to a group of predictors was quantified in the same way. This tuning index gives a lower bound on the amount of variability in the data that can be explained by the predictor (or group of predictors) of interest. Whenever a dichotomous “tuned”/“not Tuned” characterization was needed, such as in the donut plots in Figs. 5–7, we classified as “tuned” neurons that met a 5% tuning threshold for grouped predictors (Figs. 5a, 6a, 7a).

Each neuron’s fitted model provided tuning estimates for all predictors, as well as neuron-level estimates of the model kernels such as those in Figs. 5f, 6d, i and Fig S6d, S7b, e. The pathway-level kernels in Figs. 5i, 6g, l and S6g, S7h, were defined as the root-mean-square of the kernels of task-relevant neurons in each pathway, and their confidence intervals were determined by bootstrapping over the set of neurons (10,000 bootstrap samples). The pathway kernel for a certain predictor can be given an intuitive geometrical interpretation as follows: if we consider the pseudo-population vector describing the activity of all recorded (and task-relevant) neurons within a pathway, the pathway kernel for a predictor at lag *t* is an estimate of the distance at lag *t* of the population vector from its time-averaged value, after accounting for the effect of the other predictors. By construction, then, the pathway kernels can never be negative, as they capture the overall magnitude of the effect of the predictor on the neural population, rather than a specific modulation direction. The statistical significance of the difference of pathway kernels in Figs. 5i, 6g, l and S6b, g was assessed with a bootstrap test^60^, performed with the Bias-Corrected and accelerated(BCa) technique and bootstrapping over the set of neurons belonging to the two pathways (10,000 bootstrap samples).

### Optogenetics

To evaluate the behavioral role of each pathway (PL::A-DMS or PL::P-DMS), we used a Halorhodopsin-induced terminal suppression strategy (Felix-Ortiz et al., 2013). AAV5/CaMKii-NpHR3.0-eYFP (UNC vector core) or AAV5/Syn-GFP (Penn vector core) was injected bilaterally to the PL followed by bilateral implantation of custom-made optic cannulas (Thorlabs, FT200EMP, SFLC230) for pathway-specific light delivery into either A-DMS and P-DMS. The exposed internal portion of the fiber optic was completely painted by black nail polish, in an effort to reduce stray light emission above the fiber mouth. To ensure full expression of NpHR in the axonal terminal, a recovery period of at least 5 weeks was allowed after viral injection. Animals were acclimated to the fiber optic tethers for at least 5 days before any behavioral sessions. Once animals performed >200 trials/day with a relative reward stay >2 we proceeded to the optogenetic manipulation phase, the training proceeded to the light delivery stage. In the behavior task, two light delivery protocols (~530 nm light from either PrizmatixFC-LED-535-TR or Shanghai DPSS, SDL-532-100T) were used to assess temporally distinct contributions from each pathway. To prevent light-induced non-specific effect, light intensity was adjusted to 0.8–1.5 mW at the fiber end^60^. Choice epoch manipulations were continuous illumination from initiation poke to the end of the reward delivery delay following choice. Outcome epoch manipulations were continuous illumination from outcome delivery until next trial center light on. Either ΔWin-Stay or ΔLose-Stay was calculated as:

$$\triangle {WinStay}\left({ON} - {OFF}\right)={{{{{\rm{P}}}}}}({Stay}|{Win},\,{Light}\,{ON}) - {{{{{\rm{P}}}}}}({Stay}|{Win},\,{Light}\; {OFF})$$

$$\triangle {LoseStay}({ON}-{OFF})={{{{{\rm{P}}}}}}({Stay}|{Lose},\,{Light}\,{ON}) - {{{{{\rm{P}}}}}}({Stay}|{Lose},\,{Light}\,{OFF})$$

where Win or Lose indicates reward history on prior trial. Light On/Off refers to presence or absence of light illumination on previous choice epoch (Figs. 8 and S10), current choice epoch (Fig. S10) and previous outcome epoch (Figs. 8 and Fig. S11). Behavioral data were collected for multiple days to obtain enough trials (3351 ± 143 trials, mean ± SEM across animals). Unless otherwise noted, probability of reward was 85%. For Fig. S11f, g, some PL::A-DMS outcome optogenetic sessions were performed with reward probabilities of 1.0 or 0.4, applied to both ports.

### Quantification and statistical analysis

All data were analyzed with prism9.0 and custom MATLAB code, available upon request. ANOVA (repeated measures, ordinary, mixed), t-test (paired and unpaired) were performed using Prism 9.0 built-in-function. K-S tests were performed as indicated in results using *kstest2* functions in MATLAB. Kernel density estimates were performed as indicated in results using *ksdensity* function in MATLAB. Significant effects and *p*-values are indicated in the figures and legends. Choice patterns for given *Q* value were regressed using *glmfit* function in MATLAB.

### Reporting summary

Further information on research design is available in the Nature Portfolio Reporting Summary linked to this article.

## Supplementary information

Supplementary Information

Reporting Summary

## Data Availability

Source data are provided with this paper.

## Source data

Source Data

## Code availability

All code and example data related to calcium imaging encoding model is available at https://github.com/Fuccillo-Lab.
