# Supplementary material for: Distributed processing for value-based choice by prelimbic circuits targeting anterior-posterior dorsal striatal subregions in male mice
Source: Nat Commun. 2023 Apr 6;14:1920. doi: 10.1038/s41467-023-36795-4 (PMC10079960; doi:10.1038/s41467-023-36795-4)
Supplement: Supplementary file 1 — Supplementary Information [file 41467_2023_36795_MOESM1_ESM.pdf]

Fig. S1

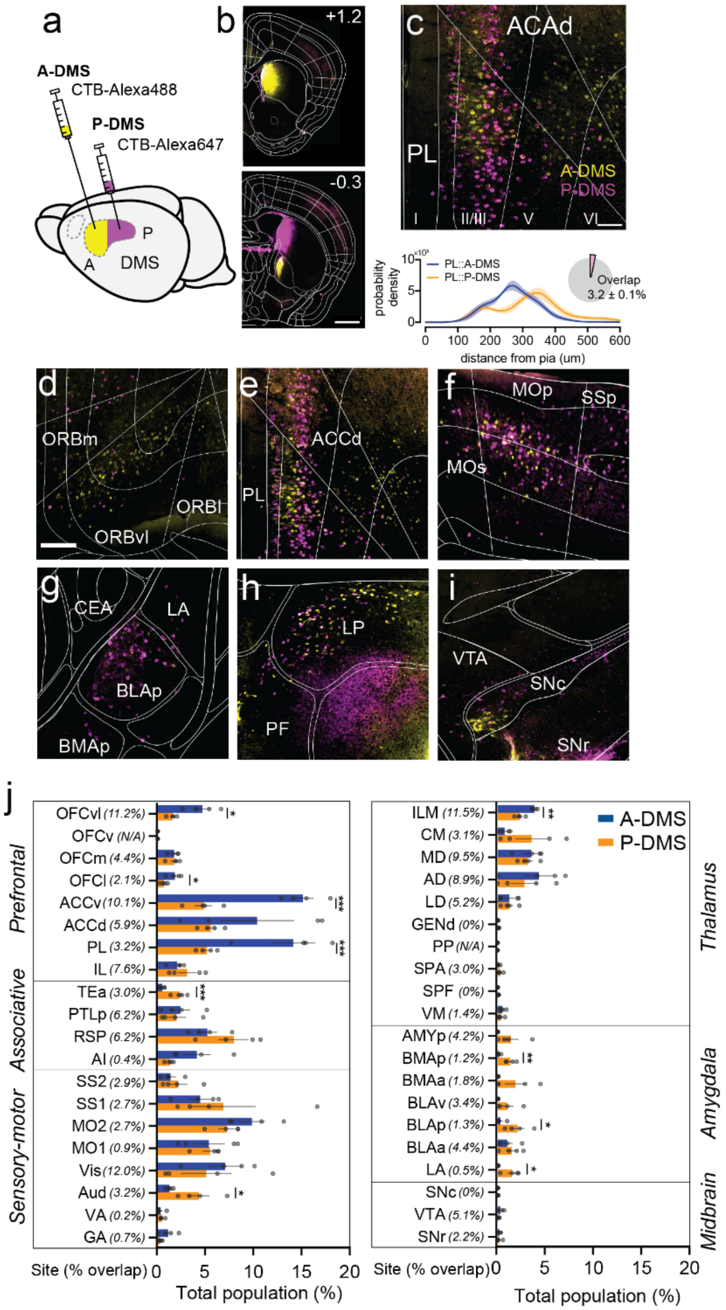

Fig S1. Assessment of brain-wide afferent inputs to anterior/posterior DMS compartments. a) Schematic showing dual retrograde tracing strategy using both Alexa488 conjugated-CTB in A-DMS (yellow) and Alexa647-conjugated CTB in P-DMS (magenta). b) Example coronal section showing injection sites (*top*: A-DMS, *bottom*: P-DMS; also note purple and yellow staining from striatal projections to GPe in bottom panel, n=4 animals). Scale bar, 1000 μm. Numbers in upper right corners indicate A/P coordinate from bregma. c) Representative image from prelimbic coronal section (*top*) and quantification of neuronal distribution(Thick line, mean; shaded area, ± SEM) from the pia (*bottom*) and overlapping population (inset). scale bar, 100 μm (n= 4 animals).

d-i) Example coronal sections of major sources of afferent inputs to A/P DMS. j) Quantification of relative proportion of labeled neurons targeting A/P DMS(mean ± SEM). Percentage number in parentheses denotes the proportion of overlapping population in brain area (OFCvl $p=0.0133$; OFCl $p=0.0278$; ACCv $p=0.0003$; PL $p=0.0079$; TEa $p=0.0034$; Aud $p=0.0337$; $n=4$ animals, two-sided unpaired t-test). *$p<0.05$, **$p<0.01$, ***$p<0.001$. Abbreviations references see Supplementary table 2.

Fig. S2

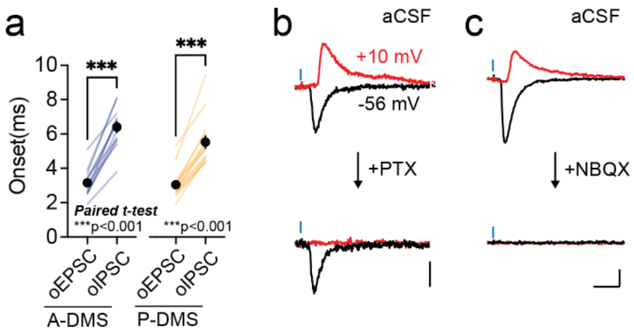

Fig S2. Temporal/Pharmacological isolation of feed-forward inhibition from PL::A/P-DMS
a) Comparison of onset latency for EPSC/IPSC from PL::A/P-DMS. Black, mean ± SEM; colored line, comparison of oEPSC and oIPSC for each cell (p=2e-8, A-DMS, n= 13cells /3 animals; p=7e-9, P-DMS, n=15 cells/3 animals, two-sided paired t-test) . b) Representative traces showing outward current (+10mV) blocked by Picrotoxin. c) Representative traces showing blockade of both direct glutamatergic inward current and di-synaptic GABAergic outward current by NBQX. Scale bar, 10ms, 50 pA.

Fig. S3

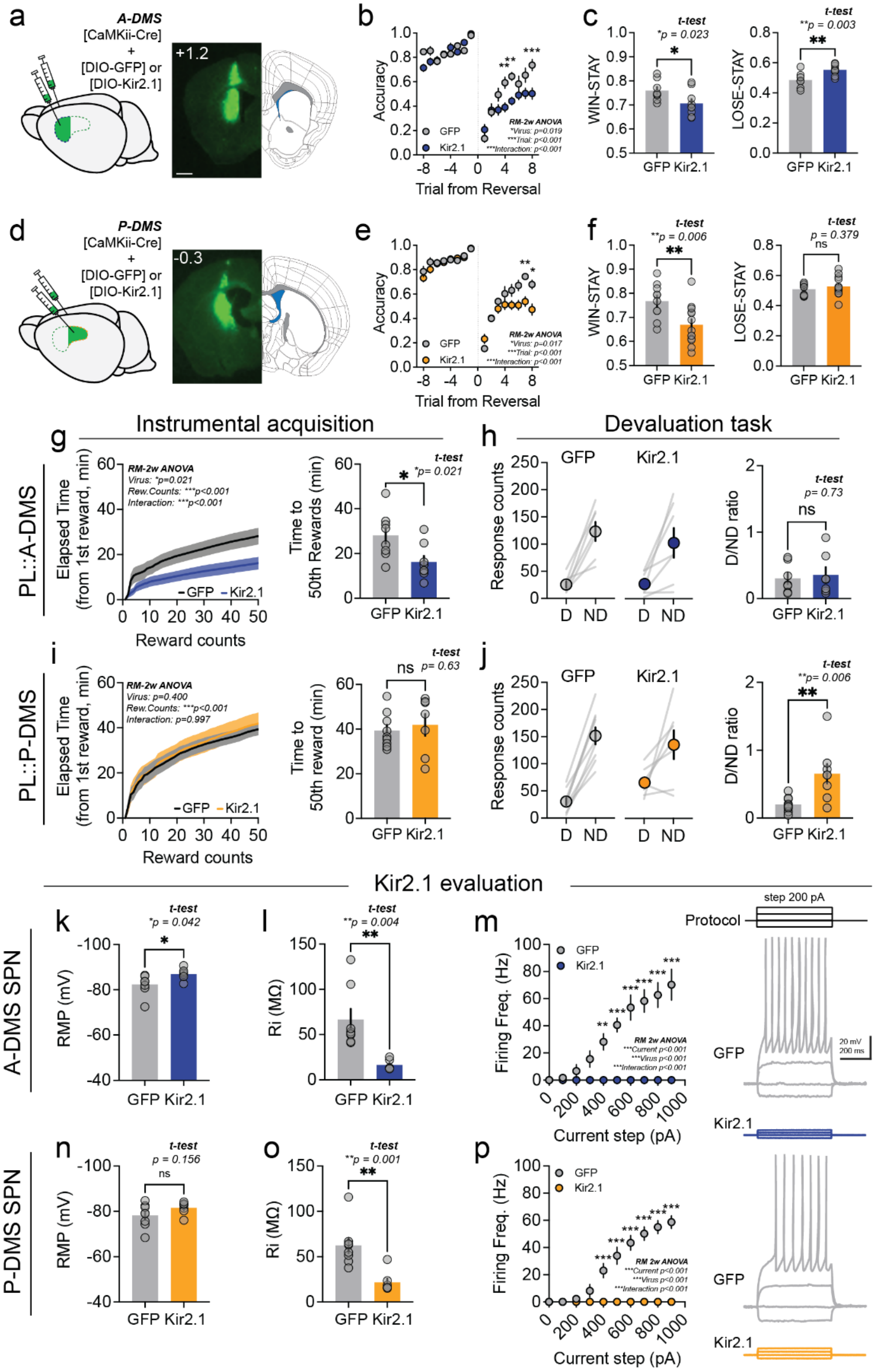

A-DMS
[CaMKii-Cre] + [DIO-GFP] or [DIO-Kir2.1]
P-DMS
[CaMKii-Cre] + [DIO-GFP] or [DIO-Kir2.1]
Accuracy
Trial from Reversal
WIN-STAY
LOSE-STAY
GFP Kir2.1
Instrumental acquisition
Devaluation task
PL::A-DMS
PL::P-DMS
Elapsed Time (from 1st reward, min)
Reward counts
Time to 50th Rewards (min)
Response counts
D/ND ratio
Kir2.1 evaluation
A-DMS SPN
P-DMS SPN
RMP (mV)
Ri (MΩ)
Firing Freq. (Hz)
Current step (pA)
Protocol
step 200 pA

Fig S3. Striatal subregion-specific inactivation reveals differential influences on flexible value-based choice. a) Schematic showing surgery for bilateral A-DMS suppression (*left*) and representative expression image (*right,* n= 8/10 animals). Number denotes A-P coordinates from bregma. Scale bar, 1000 μm. b) Comparison of accuracy(mean ± SEM) aligned by contingency shift (trial 0) between GFP and Kir2.1 in A-DMS (t5 p=0.0051, t6 p=0.004, t8 p=0.0002, A-DMS[GFP], n= 8 animals; A-DMS[Kir2.1], n= 10 animals, Šidák multiple comparison test). **p<0.01, ***p<0.001. c) Proportion of Win-stay (*right,* p=0.023) and Lose-stay (*left,* p=0.003) choices(mean ± SEM) in mice where A-DMS was infected with either GFP or Kir2.1(A-DMS[GFP], n= 8 animals; A-DMS[Kir2.1], n= 10 animals, two-sided unpaired t-test). *p<0.05, **p<0.01. d-f) Same as a, but for P-DMS (n=10/11 animals). e) Comparison of accuracy(mean ± SEM) aligned by contingency shift(t7 p=0.0075, t8 p=0.0362; P-DMS[GFP], n= 10 animals; P-DMS[Kir2.1], n=13 animals, Šidák multiple comparison test). *p<0.05, **p<0.01. f) Proportion of Win-stay (*right,* p=0.006) and Lose-stay (*left,* p=0.379) choices(mean ± SEM) in mice. P-DMS[GFP], n= 10 animals; P-DMS[Kir2.1], n= 13 animals, two-sided unpaired t-test). **p<0.01. g, i) Comparison of instrumental acquisition rate between GFP and Kir2.1 overexpression in total elapsed time per reward count (left, Šidák post-hoc analysis) and elapsed time for 50th reward (right) (p=0.021, A-DMS[GFP], n= 8 animals; A-DMS[Kir2.1], n= 8 animals; p=0.63, P-DMS[GFP], n= 9 animals; P-DMS[Kir2.1], n= 7 animals, two-sided unpaired t-test). *p<0.05, ***p<0.001. h, j) Devaluation task. Number of responses in extinction conditions after 1h pre-exposure to D (same reward as operant training) or ND (different reward) (left) and comparison of D/ND ratio between GFP and Kir2.1 overexpression (p=0.73, A-DMS[GFP] n= 7, A-DMS[Kir2.1] n= 7; p=0.006, P-DMS[GFP] n= 9, P-DMS[Kir2.1] n= 8, two-sided unpaired t-test). **p <0.01. k-m) Comparison of passive and active membrane properties recorded from GFP (n=8 cells) or Kir2.1(n= 6 cells) expressing spiny neuron in A-DMS(mean ± SEM) – k) Resting membrane potential (p=0.042, two-sided unpaired t-test). l) Input resistance (p=0.004, two-sided unpaired t-test). m) firing frequency in response to increasing current injections (left, 400pA p=0.0087, 500pA p=3e-5, 600pA p=2e-8, 700pA p=1e-9, 800pA p=8e-11, 900pA<1e-12; Šidák multiple comparison test) and representative trace with correspond current injection protocol (right). *p<0.05, **p<0.01, ***p<0.001. n-p) Comparison of passive and active membrane properties recorded from GFP (n= 8 cells) or Kir2.1(n= 7 cells) expressing spiny neuron in P-DMS (mean ± SEM) – n) Resting membrane potential(p=0.156, two-sided unpaired t-test). o) Input resistance(p=0.001, two-sided unpaired t-test). p) firing frequency in response to increasing current injections (left, 400pA p=4e-5, 500pA p=8e-10, 600pA p<1e-12, 700pA p<1e-12, 800pA p<1e-12, 900pA<1e-12; Šidák multiple comparison test) and representative trace with correspond current injection protocol (right) **p<0.01, ***p<0.001.

Fig. S4

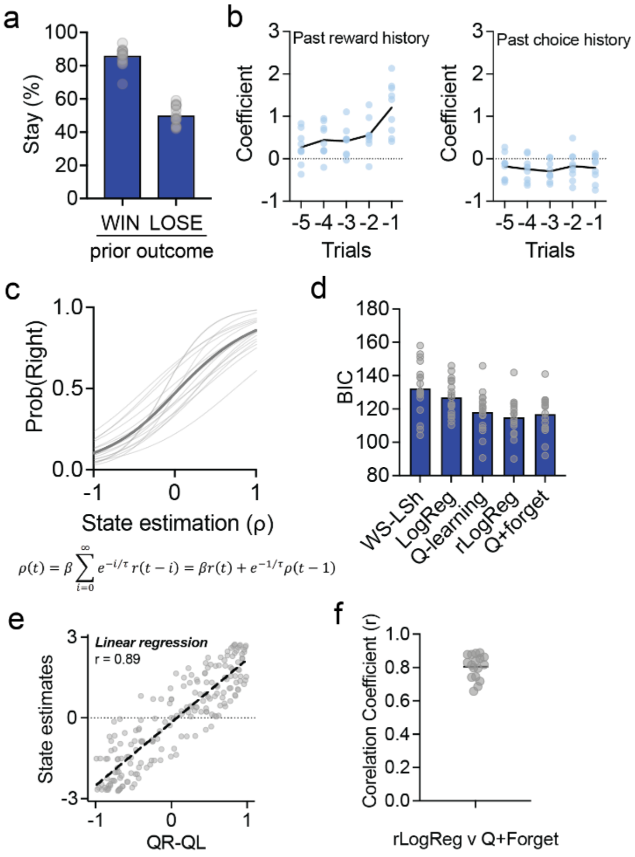

$$\rho(t) = \beta \sum_{i=0}^{\infty} e^{-i/\tau} r(t-i) = \beta r(t) + e^{-1/\tau}\rho(t-1)$$

Fig S4. Modeling of choice behavior in value-based task. a) Percentage of Win-Stay and Lose-Stay choices(mean ± SEM) from all sessions (n= 17 animals). b) Regression coefficients from logistic regression (LogReg) behavioral model quantifying the impact of past 5 trial outcomes (*left*) and choices (*right*) on current choice. c) Probability of right choice as a function of the certainty of 'right rewarded' state estimation from rLogReg model. Mean curve (thick grey line) and individual animal replicates (thin grey line). d) BIC comparison(mean ± SEM) from five behavior models of choice behavior (WS-LSh, WinStay-LoseShift; LogReg, Logistic Regression; Q-learning, standard q-learning model; rLogReg, recursive Logistic Regression; Q+forget, q-learning model with forgetting for unchosen choice, n= 17 biologically independent animals). e) Single animal trial-by-trial correlation of QR-QL (ΔQ) from Q+forget model and state estimates from rLogReg model(grey dot, trial state estimates for a given QR-QL; black dotted line, prediction from linear regression, r=0.89, p<0.0001). f) Correlation coefficient between value differences and state-estimates for all recorded animals.

Fig. S5

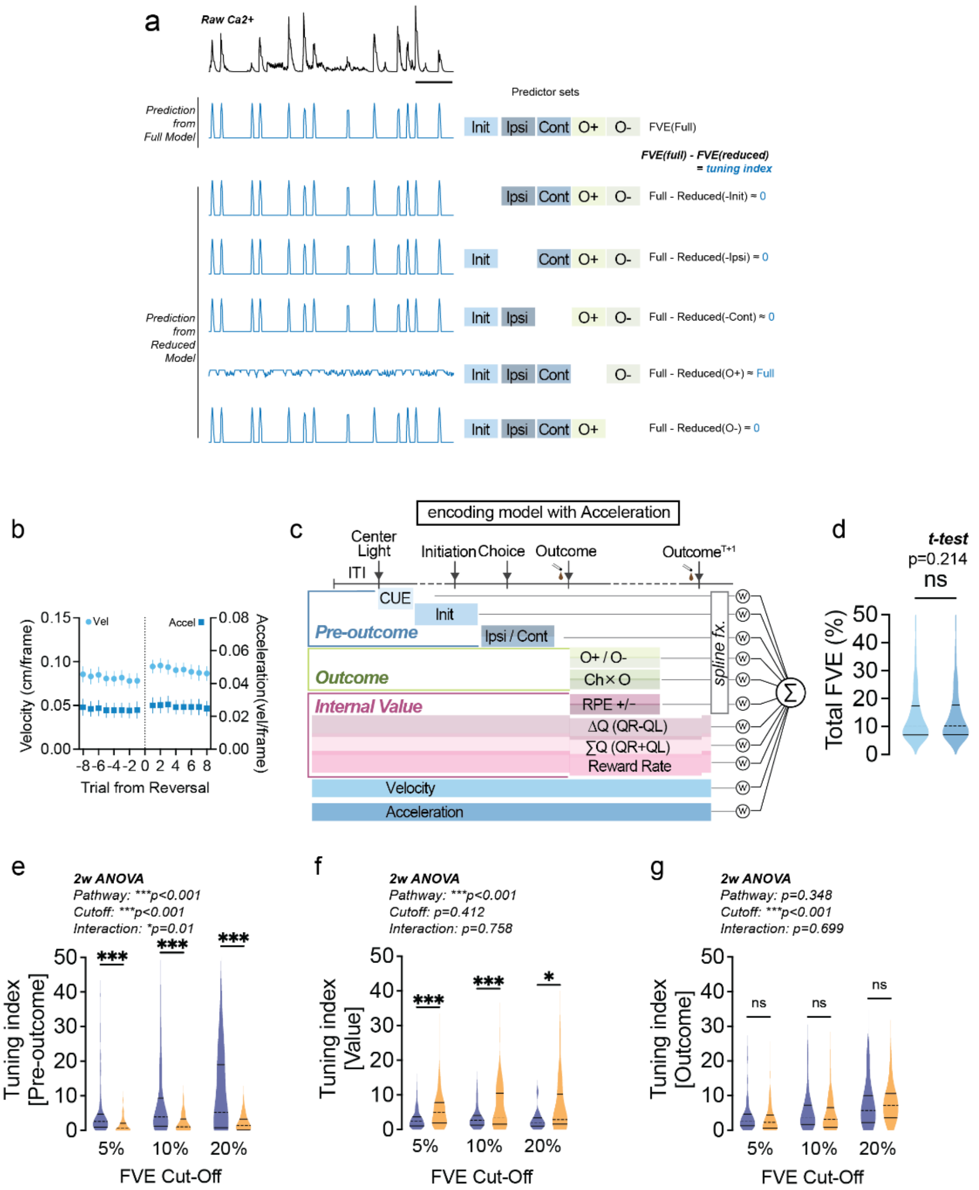

Fig S5. Illustration of tuning index calculations, encoding model with acceleration predictors and effects of varying FVE cut-offs. a) Illustration of how tuning indices were calculated. (Top) Raw calcium signal and the output prediction from the full model (individual predictors shown at right). (Bottom) The model output for partial models with a single predictor missing (shown at right). b) Trial averaged velocity(mean ± SEM) and acceleration plot aligned by reversal (n= 17 animals). c) Design matrix structure for modified neural encoding model with added acceleration predictor. d) A comparison of total FVE(Shaded area, Kernel probability density; solid line, quartile; dotted line, median) between current model and model with acceleration predictor (p= 0.214, n= 253 pairs, two-sided paired t-test). e-g)Summary tuning index (Shaded area, Kernel probability density; solid line, quartile=7.046,17.29/7.115,17.64; dotted line, median=10.11/10.16) for e) pre-outcome(5%

p=1e-5 quartile=0.9899,4.791/0,2.096 median=2.666/0.7354, 10% p=7e-7 quartile=1.217,9.353/0,3.298 median=3.856/1.050, 20% p=5e-7 quartile=0.8327,19.0/0.1342,3.280 median=5.194/1.472), f) value(5% p=8e-6 quartile=1.052,3.685/1.939,7.831 median=2.410/4.972, 10% p=1e-4 quartile=1.283,4.061/1.551,10.48 median=2.671/3.568, 20% p=0.0129 quartile=0.9648,3.342/1.589,10.17 median=2/2.884) and g) outcome(5% p=0.4744 quartile=1.332,4.708/0.5896,4.394 median=2.740/2.318, 10% p=0.5081 quartile=1.678,7.228/0.8459,6.578 median=3.771/3.176, 20% p=0.9962 quartile=2.228,10.03/3.611,10.60 median=5.773/7.130) modulation for different FVE cut-off thresholds. (5% n=104/154, 10% n=56/75, 20% n=28/24; PL::A-DMS/PL::P-DMS respectively, Šidák post-hoc analysis). *p<0.05, ***p<0.001.

Fig. S6

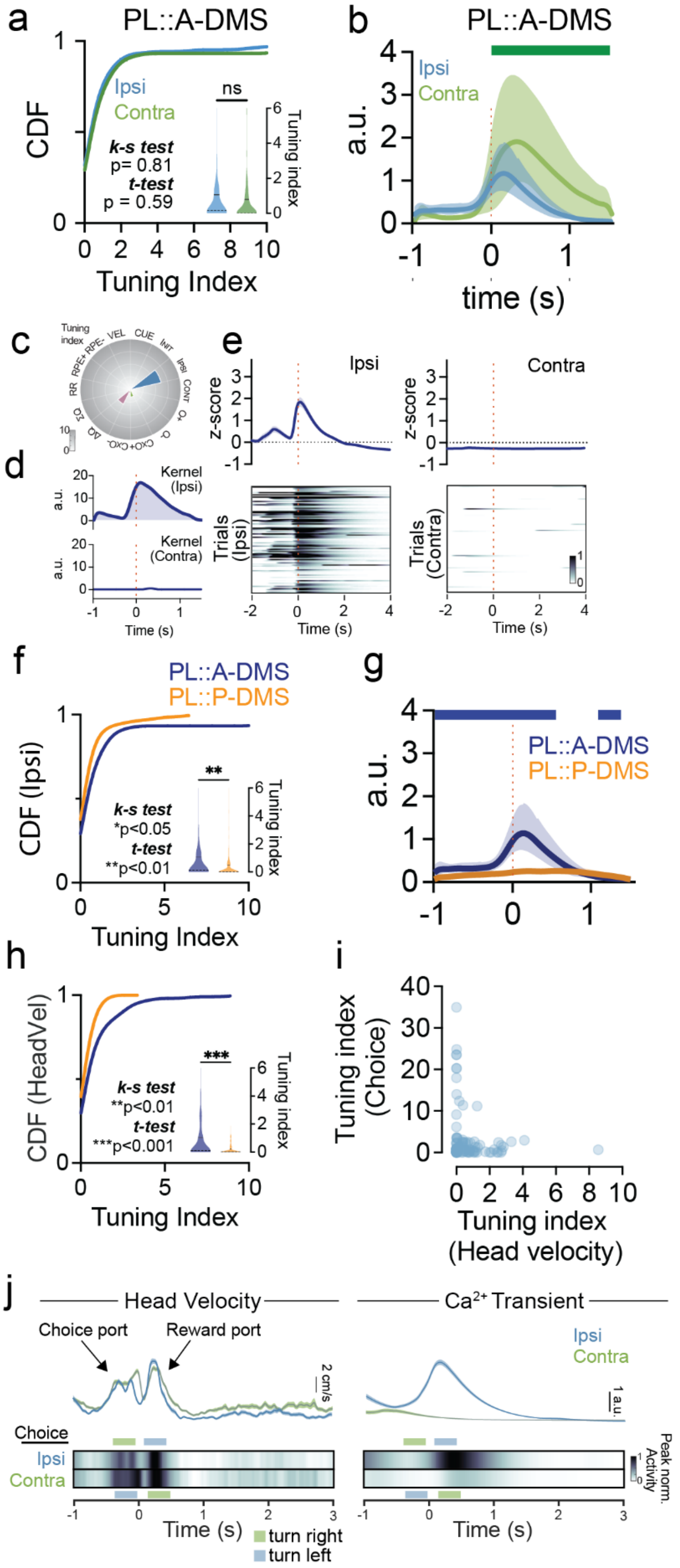

a
PL::A-DMS
CDF
Ipsi
Contra
k-s test
p= 0.81
t-test
p = 0.59
ns
Tuning index
Tuning Index
b
PL::A-DMS
Ipsi
Contra
a.u.
time (s)
c
Tuning index
d
Kernel (Ipsi)
Kernel (Contra)
a.u.
Time (s)
e
Ipsi
Contra
z-score
Trials (Ipsi)
Trials (Contra)
Time (s)
f
PL::A-DMS
PL::P-DMS
CDF (Ipsi)
k-s test
*p<0.05
t-test
**p<0.01
Tuning index
Tuning Index
g
PL::A-DMS
PL::P-DMS
a.u.
h
CDF (HeadVel)
k-s test
**p<0.01
t-test
***p<0.001
Tuning index
Tuning Index
i
Tuning index (Choice)
Tuning index (Head velocity)
j
Head Velocity
Ca2+ Transient
Choice port
Reward port
2 cm/s
Ipsi
Contra
1 a.u.
Choice
Peak norm. Activity
Time (s)
turn right
turn left

Fig S6. PL::A-DMS circuit encodes both choice and movement.

a) Comparison of cumulative distributions ($p=0.81$, Two-sided Kolmogorov-Smirnov test) and average tuning index (insets; Shaded area, Kernel probability density; solid line, quartile=0,1.059/0,0.7890; dotted line, median=0.1542/0.0101) of Ipsi/Contra choice predictors from PL::A-DMS ($p=0.59$, PL::A-DMS, $n=104$ cells, Two-sided unpaired t-test). b) Comparison of model inferred ipsi/contra choice kernels on average for PL::A-DMS pathways. Solid line, root-mean-squared; shaded area, 95% confidence interval. Colored-bar on top indicates significant mean-displacement on each timepoint between pathways. c) Tuning plot showing representative ipsilateral choice tuned neuron from PL::A-DMS. d) Encoding model inferred kernels corresponding to ipsi (*top*) and contra (*bottom*) choice. e) PETH (*top*) and trial-by-trial neuronal activity (*bottom*) aligned by Ipsi (*left*)/Contra (*right*) choice. Solid line, mean; shaded area, SEM. f) Comparison of cumulative distribution($p=0.011$, Two-sided Kolmogorov-Smirnov test) and average tuning index (insets; Shaded area, Kernel probability density; solid line=0,1.059/0,0.4634, quartile; dotted line, median=0.1542/0) of ipsilateral choice tuned neurons in both PL::A/P-DMS pathways ($p=0.002$, PL::A-DMS, $n=104$ cells; PL::P-DMS, $n=154$ cells, Two-sided unpaired t-test). **$p<0.01$. g) Comparison of model inferred ipsilateral choice kernels on average for both PL::DMS pathways. Solid line, root-mean-squared; shaded area, 95% confidence interval. Colored-bar on top indicates significant mean-displacement on each timepoint between pathways. h) Comparison of cumulative distributions($p=0.002$, Two-sided Kolmogorov-Smirnov test) and average tuning index (insets; Shaded area, Kernel probability density; solid line, quartile=0,1.036/0,0.2374; dotted line, median=0.0882/0.) of head velocity tuned neurons from PL::A/P-DMS pathways($p=2e-6$, PL::A-DMS, $n=104$ cells; PL::P-DMS, $n=154$ cells, Two-sided unpaired t-test). ***$p<0.001$. i) Scatter plot of head velocity/choice tuning index from task tuned neurons. Blue dot denotes each neuron from PL::A-DMS. j) Comparison of head velocity (*left*) and Ca^{2+} transients (*right*) for representative ipsilateral choice encoding neuron. Single-trial (top) and mean values(bottom) of velocity and Ca2+ transient. Note that both ipsi and contra choice have the opposite motions when returning to the reward port (green bar, right turn; blue bar, left turn). Further note that the Ca^{2+} transient selectively response to ipsilateral choice, not ipsilateral head movement. Solid line, mean; shaded area, ±SEM.

Fig. S7

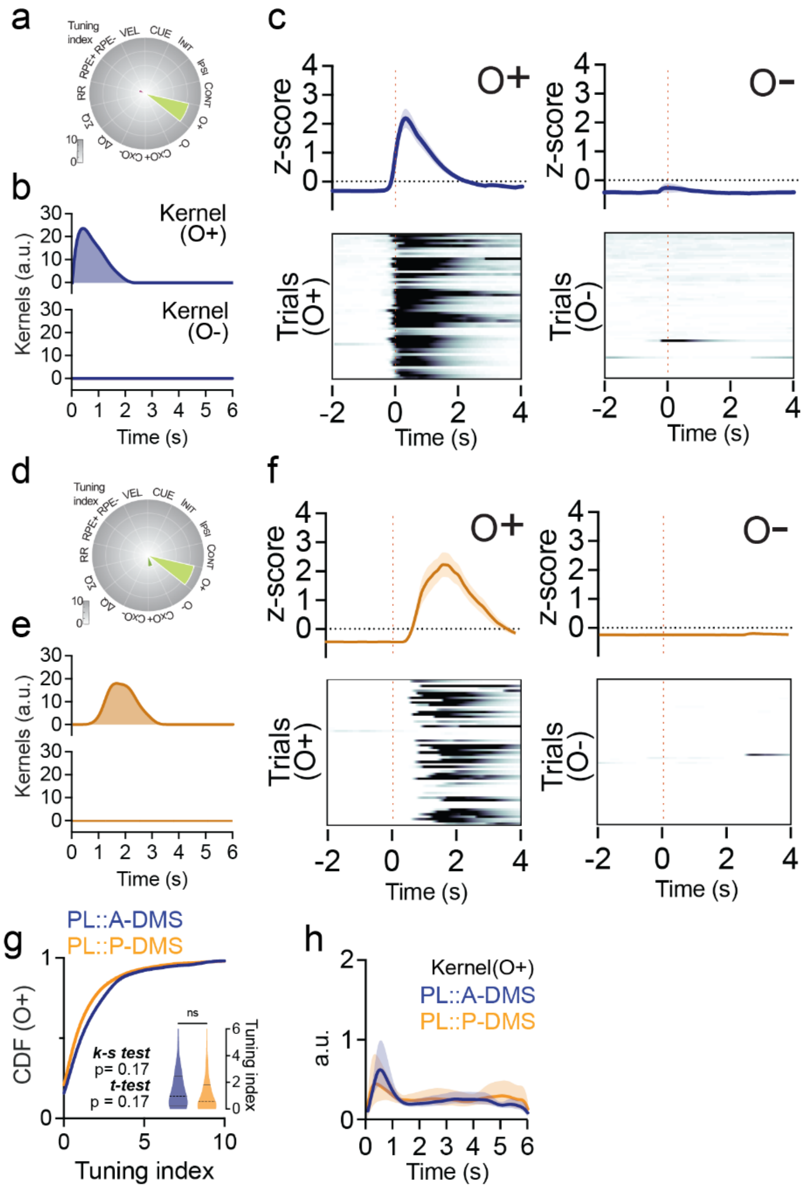

Fig S7. Detailed analysis of outcome encoding. a) Tuning plot showing representative O+ tuned neuron from PL::A-DMS. b) Encoding model inferred kernels corresponding to O+ (*top*) and O- (*bottom*). c) z-scored PETH (*top*) and trial-by-trial normalized neuronal activity (*bottom*) corresponding to O+ (*left)* and O-(*right*) from PL::A-DMS. Solid line denotes mean; shaded area denotes SEM. d-f) Same as a, but O+ encoding neuron from PL::P-DMS circuits. g) Comparison of cumulative distribution(p=0.1733, Two-sided Kolmogorov-Smirnov test) and average tuning index (insets; Shaded area, Kernel probability density; solid line, quartile=0.2347,2.473/0,1.726; dotted line, median=0.9635/0.5879.) of O+ tuned neurons in both PL::A/P-DMS pathways(p=0.1718, PL::A-DMS, n=104 cells; PL::P-DMS, n=154 cells, Two-sided unpaired t-test). h) Comparison of model inferred O+ kernels on average for both PL-DMS

pathways. Solid line, root-mean-squared; shaded area, ±95% confidence interval. Colored-bar on top indicates significant mean-displacement on each timepoint between pathways.

Fig. S8

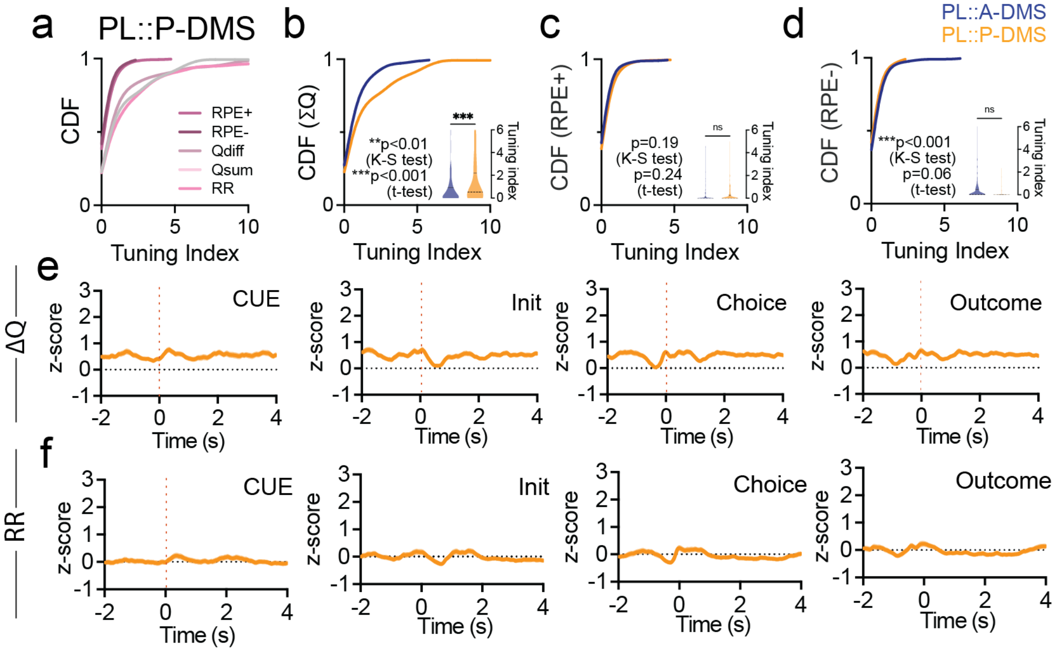

Fig S8. Detailed analysis of internal value neural coding. a) Cumulative distributions for individual internal predictors from PL::P-DMS. b-d) Pathway comparison using cumulative distributions and mean tuning index (inset) of b) ∑Q (p=0.003, two-sided Kolmogorov-Smirnov test; Shaded area, Kernel probability density; solid line, quartile=0,0.9066/0,2.206; dotted line, median=0.2777/0.5116 ;p=6e-4, PL::A-DMS, n=104 cells; PL::P-DMS, n=154 cells, Two-sided unpaired t-test), c) RPE+(p=0.19, two-sided Kolmogorov-Smirnov test; solid line, quartile=0,0.0773/0,0.2629; dotted line, median=0/0; p=0.24, PL::A-DMS, n=104 cells; PL::P-DMS, n=154 cells, Two-sided unpaired t-test), d) RPE-+(p=5e-4, two-sided Kolmogorov-Smirnov test; solid line, quartile=0,0.4429/0,0.0347; dotted line, median=0.011/0; p=0.06, PL::A-DMS, n=104 cells; PL::P-DMS, n=154 cells, Two-sided unpaired t-test) tuning from task-tuned neurons. *p<0.05, **p<0.01, ***p<0.001. e) PETH for ΔQ encoding neuron corresponding to Fig. 7d, aligned to behavioral events (right corner). f) PETH for RR encoding neuron corresponding to Fig. 7h, aligned to behavioral events name in right top corner.

Fig. S9

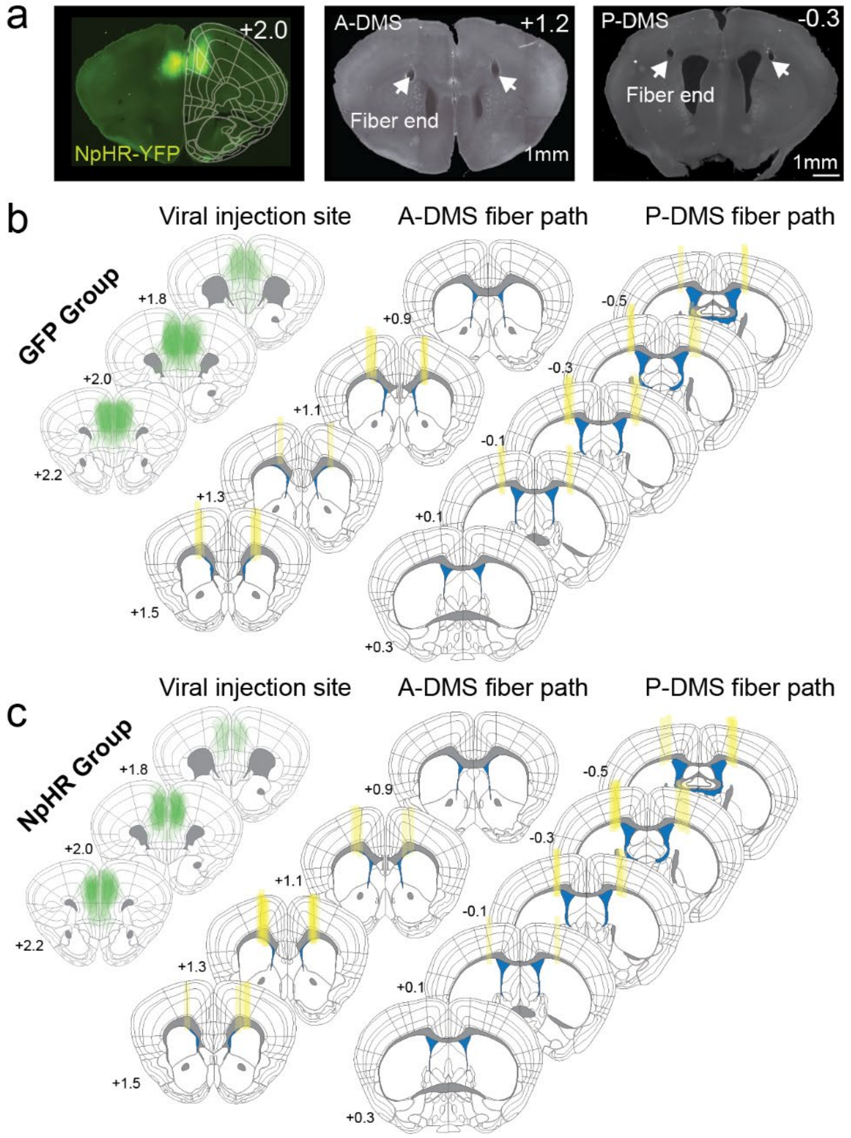

Fig S9. Injection sites for optogenetic experiments. a) Representative images from injection site (left, PL) and fiber implanted site (middle, A-DMS; right, P-DMS) b) Brain atlas images showing GFP control virus injection site (left) and bilateral fiber implant site from A-DMS (center) and P-DMS groups (right). c) same as b, but NpHR virus injected group.

Fig. S10

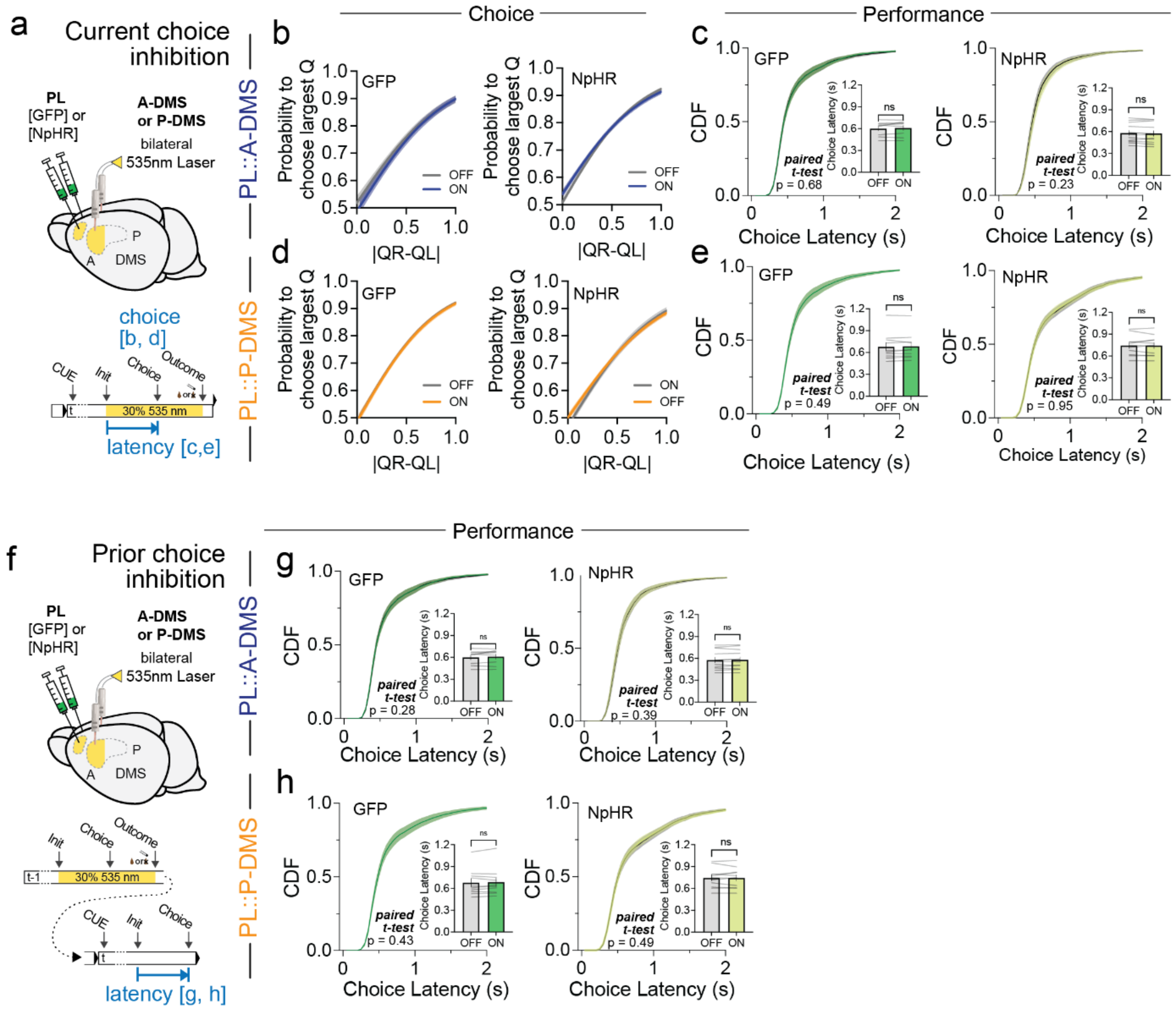

Fig S10. Effects of optogenetic suppression during choice epoch on behavior in current and subsequent trials. a) Schematic showing surgery for pathway specific suppression and light delivery protocol for current choice inhibition. b) Comparison of probability to choose largest Q value for a given Q value differences between GFP (*left*) and NpHR (*right*) groups, when light was delivered in the current choice epoch to A-DMS. Solid line, mean; shaded area, ±SEM. c) Cumulative distribution of choice latencies and comparison of average choice latency (inset, mean ± SEM) between GFP (*left,* p=0.68) and NpHR (*right, p=0.23*) from PL::A-DMS terminal illumination (GFP/NpHR n= 9/12 animals, two-sided paired t-test). Solid line, mean; shaded area, ±SEM. d-e) same as b-c, but for PL::P-DMS (left, p=0.49; right p=0.95; GFP/NpHR n=10/11 animals, two-sided paired t-test). f) same as a, but prior choice inhibition protocol. g) Comparison of cumulative distribution and average(insets, mean ± SEM) of choice latency in light ON/OFF on prior trials for PL::A-DMS circuits infected with either GFP(*left, p=0.28*) or NpHR(*right, p=0.39*). Solid line, mean; shaded area, ±SEM (GFP/NpHR n= 9/12 animals, two-sided paired t-test). h)

same as g, but for PL::P-DMS (left, $p=0.43$; right, $p=0.49$; GFP/NpHR $n=10/11$ animals, two-sided paired t-test).

Fig. S11

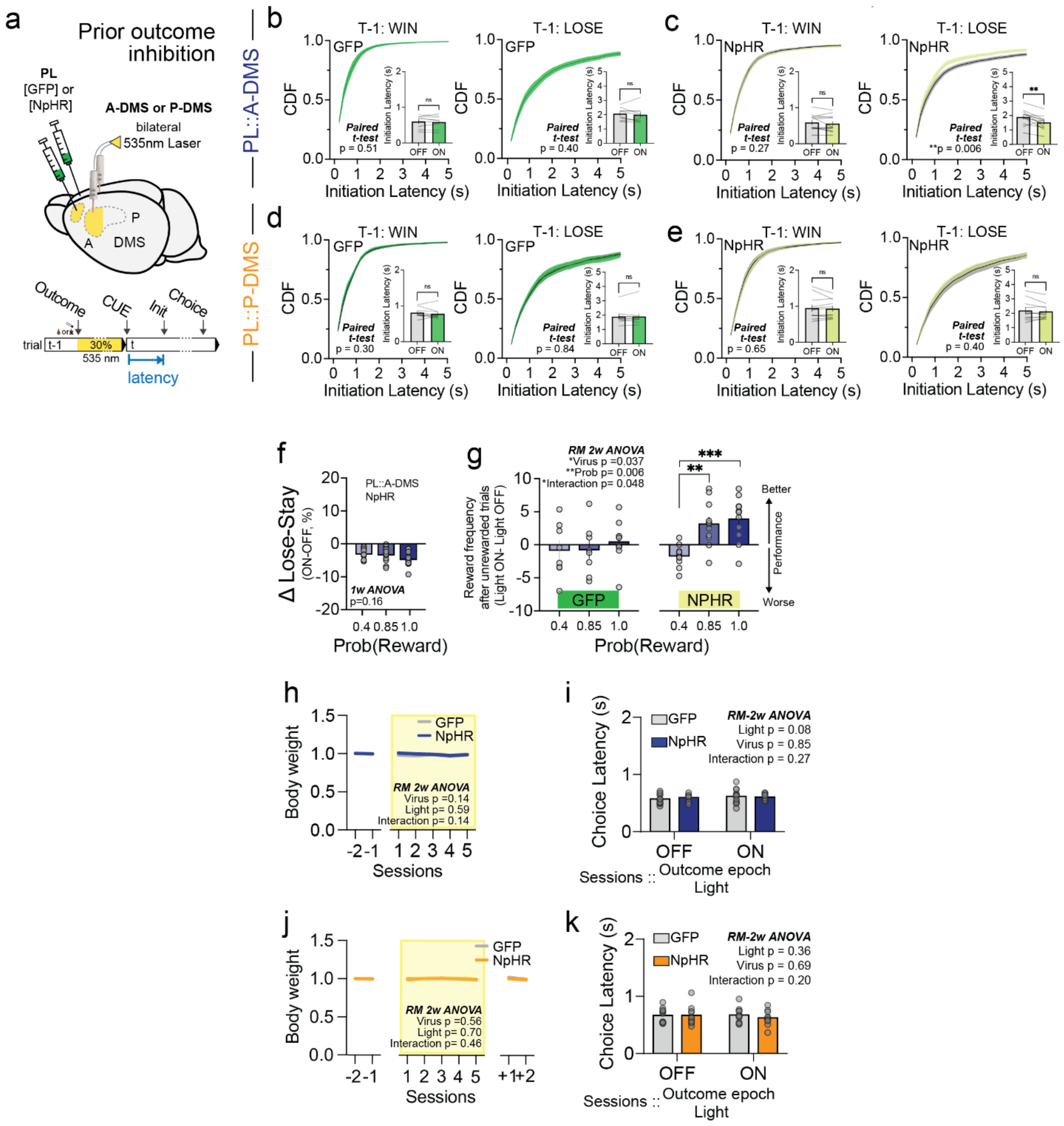

Fig S11. Effects of optogenetic suppression during outcome epoch on behavior in subsequent trials and broad effect on session. a) Schematic showing surgery for pathway specific suppression and light delivery protocol for prior outcome inhibition. b-c) Comparison of cumulative distribution (Black line, mean; shaded area, ±SEM) and average(insets, mean ± SEM) of initiation latency on prior WIN(*left*)/LOSE(*right*) in light ON/OFF for PL::A-DMS circuits infected with GFP(b; left, p=0.51; right, p=0.40) or NpHR(c; left, p=0.27; right, p=0.006) (GFP/NpHR n= 9/12 animals, two-sided paired t-test). **p<0.01. d-e) Comparison of cumulative distribution (Black

line, mean; shaded area, ±SEM) and average(insets) of initiation latency on prior WIN(*left*)/LOSE(*right*) in light ON/OFF for PL::P-DMS circuits infected with GFP(d; left, p=0.30; right, p=0.84) or NpHR(e; left, p=0.65; right, p=0.40) (GFP/NpHR n=10/11 animals, two-sided paired t-test). f) Comparison of ∆Lose-Stay(mean ± SEM) consequences for PL::A-DMS inhibition across a range of reward probability paradigms (P_{rew}=1.0/0.85/0.4 n=11/12/10 animals, respectively). g) Percentage changes of reward frequency(mean ± SEM) after unrewarded trial (Light ON – Light OFF) between across a range of reward probability paradigms from GFP and NpHR (Pr0.4-Pr0.85 p=0.0028, Pr0.4-Pr1.0 p=0.0006) in PL::A-DMS (p=GFP/NpHR n=8/11 animals, Šidák multiple comparison test). **p<0.01, ***p<0.001. h) Normalized body weight per session in sessions without and with outcome optogenetic inhibition of PL::A-DMS pathway in a random 30% of trials (yellow box). Solid line, mean; shaded area, ±SEM. i) Comparison of choice latency(mean ± SEM) between sessions with (ON) or without (OFF) outcome epoch illumination of PL::A-DMS circuits from either GFP or NpHR group (GFP/NpHR n=9/12 animals). j-k) same as h, k but for PL::P-DMS (GFP/NpHR n=10/11 animals).

Supplemental Figure 12

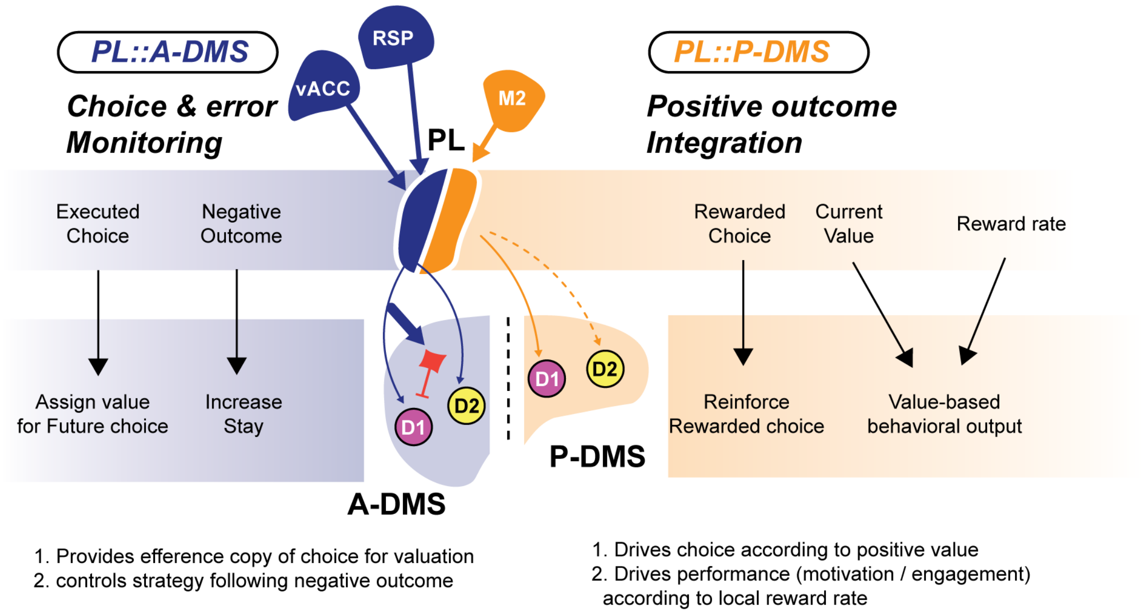

Fig S12. Model of PL::A/P-DMS circuit architecture and diverged role.

Predictor	Window size PRE (s)	Window size POST (s)	Number of splines
start cue (CUE)	0	2	12
Self-initiation (Init)	2	1	12
Choice LEFT (Contra)	1	1.5	20
Choice RIGHT (Ipsi)	1	1.5	20
Outcome NEGATIVE (O-)	0	6	20
Outcome POSITIVE (O+)	0	6	20
Choice RIGHT x Outcome NEGATIVE (Rt x O-)	0	6	20
Choice RIGHT x Outcome POSITIVE (Rt x O+)	0	6	20
Reward prediction error (RPE), positive (RPE+)	0	6	20
Reward prediction error (RPE), negative (RPE-)	0	6	20
Reward rate (RR)	(continuous predictor)		
Q(LEFT) + Q(RIGHT) (ΣQ),	(continuous predictor)		
Q(LEFT) - Q(RIGHT) (ΔQ)	(continuous predictor)		
Head Velocity	(continuous predictor)		
Acceleration (Fig. S5 c, d)	(continuous predictor)		

Supplementary Table 1. Details on model predictors. Window size PRE/POST indicates the extent of the kernel window before/after the event to which the predictor is tethered. N of splines is the size of the spline basis used for the predictor. The basis functions are cubic B-splines. The spline knots are placed at regular intervals within the kernel window; additionally, four knots are placed at each end of the window to enable flexibility in the value of the kernel and all its derivatives at the endpoints. The degree of the splines and the number of the knots determines the number of elements in the basis spline set, reported in the fourth column.

Agranular insular area	AI	Lateral amygdalar nucleus	LA
Anterior cingulate area	ACC	Lateral group of the dorsal thalamus	LAT
Anterior cingulate area/ dorsal part	ACCd	Medial group of the dorsal thalamus	MD
Anterior cingulate area/ ventral part	ACCv	Midline group of the dorsal thalamus	CM
Anterior group of the dorsal thalamus	AD	Orbital area	OFC
Auditory areas	AUD	Paraventricular nucleus of the thalamus	PVT
Basolateral amygdalar nucleus	BLA	Peripeduncular nucleus	PP
Basolateral amygdalar nucleus/ anterior part	BLAa	Posterior amygdalar nucleus	AMYp
Basolateral amygdalar nucleus/ posterior part	BLAp	Posterior parietal association areas	PTLp
Basolateral amygdalar nucleus/ ventral part	BLAv	Prelimbic area	PL
Basomedial amygdalar nucleus	BMA	Primary motor area	M1
Basomedial amygdalar nucleus/ anterior part	BMAa	Primary somatosensory area	S1
Basomedial amygdalar nucleus/ posterior part	BMAp	Reticular nucleus of the thalamus	RT
Claustrum	CLA	Retrosplenial area	RSP
Epithalamus	EPI	Secondary motor area	M2
Geniculate group/ dorsal thalamus	GENd	Subparafascicular area	SPA
Geniculate group/ ventral thalamus	GENv	Subparafascicular nucleus	SPF
Globus pallidus/ external segment	GPe	Substantia nigra/ compact part	SNc
Globus pallidus/ internal segment	GPi	Substantia nigra/ reticular part	SNr
Gustatory areas	GU	Supplemental somatosensory area	S2
Hippocampal formation	HPF	Temporal association areas	TEa
Infralimbic area	IL	Ventral group of the dorsal thalamus	VM
Intralaminar nuclei of the dorsal thalamus	ILM	Ventral tegmental area	VTA
		Visceral area	VIC

Supplementary Table 2. Abbreviation of brain area.
