# Supplementary material for: Distributed processing for value-based choice by prelimbic circuits targeting anterior-posterior dorsal striatal subregions in male mice
Source: Nat Commun. 2023 Apr 6;14:1920. doi: 10.1038/s41467-023-36795-4 (PMC10079960; doi:10.1038/s41467-023-36795-4)
Supplement: Supplementary file 2 — Reporting Summary [file 41467_2023_36795_MOESM2_ESM.pdf]

nature portfolio

Corresponding author(s): Marc V. Fuccillo

Last updated by author(s): 2023/2/9

Reporting Summary

Nature Portfolio wishes to improve the reproducibility of the work that we publish. This form provides structure for consistency and transparency in reporting. For further information on Nature Portfolio policies, see our Editorial Policies and the Editorial Policy Checklist.

Statistics

For all statistical analyses, confirm that the following items are present in the figure legend, table legend, main text, or Methods section.

n/a	Confirmed	
☐	☒	The exact sample size (*n*) for each experimental group/condition, given as a discrete number and unit of measurement
☐	☒	A statement on whether measurements were taken from distinct samples or whether the same sample was measured repeatedly
☐	☒	The statistical test(s) used AND whether they are one- or two-sided<br>*Only common tests should be described solely by name; describe more complex techniques in the Methods section.*
☒	☐	A description of all covariates tested
☐	☒	A description of any assumptions or corrections, such as tests of normality and adjustment for multiple comparisons
☐	☒	A full description of the statistical parameters including central tendency (e.g. means) or other basic estimates (e.g. regression coefficient) AND variation (e.g. standard deviation) or associated estimates of uncertainty (e.g. confidence intervals)
☐	☒	For null hypothesis testing, the test statistic (e.g. F, t, r) with confidence intervals, effect sizes, degrees of freedom and P value noted<br>*Give P values as exact values whenever suitable.*
☒	☐	For Bayesian analysis, information on the choice of priors and Markov chain Monte Carlo settings
☒	☐	For hierarchical and complex designs, identification of the appropriate level for tests and full reporting of outcomes
☒	☐	Estimates of effect sizes (e.g. Cohen's d, Pearson's r), indicating how they were calculated

Our web collection on statistics for biologists contains articles on many of the points above.

Software and code

Policy information about availability of computer code

Data collection	Electrophysiology data were collected using recording artist software(https://github.com/rgerkin/recording-artist) wrapped in Igor pro 7 (Wavemetrics Inc)<br>UCLA miniscope V3 was assembled in the lab. UCLA miniscope data was obtained using UCLA miniscope data acquisition software (http://www.miniscope.org)<br>Behavior box was purchased from Sanwork LLC (http://sanworks.io, Mouse Behavior Box r2), and all behavior data were collected using Bpod acquisition code(v1.6, https://github.com/sanworks/Bpod_Gen2) wrapped in Matlab (2016b, Mathworks).
Data analysis	Brain mapping data were analyzed using NeuroInfo Suite(v2021) which registered individual slices to the Alle Institute reference brain atlas (Allen mouse common coordinate framework; CCFv3)<br>Electrophysiology data were analyzed using Igor pro 7 (Wavemetrics Inc) and Matlab (2018b, Mathworks).<br>Behavioral model and Encoding model were analyzed in custom-built Matlab code (2018b, Mathworks).<br>Animal tracking were captured by DeepLabcut software(Version 2.2.0.6)<br>Graph were designed using Graphpad Prism9.0

For manuscripts utilizing custom algorithms or software that are central to the research but not yet described in published literature, software must be made available to editors and reviewers. We strongly encourage code deposition in a community repository (e.g. GitHub). See the Nature Portfolio guidelines for submitting code & software for further information.

Data

Policy information about availability of data

All manuscripts must include a data availability statement. This statement should provide the following information, where applicable:

- Accession codes, unique identifiers, or web links for publicly available datasets
- A description of any restrictions on data availability
- For clinical datasets or third party data, please ensure that the statement adheres to our policy

All data and codes that support the findings of this study for this study is accessible.

Human research participants

Policy information about studies involving human research participants and Sex and Gender in Research.

Reporting on sex and gender	n/a
Population characteristics	n/a
Recruitment	n/a
Ethics oversight	n/a

Note that full information on the approval of the study protocol must also be provided in the manuscript.

Field-specific reporting

Please select the one below that is the best fit for your research. If you are not sure, read the appropriate sections before making your selection.

☒ Life sciences ☐ Behavioural & social sciences ☐ Ecological, evolutionary & environmental sciences

For a reference copy of the document with all sections, see nature.com/documents/nr-reporting-summary-flat.pdf

Life sciences study design

All studies must disclose on these points even when the disclosure is negative.

Sample size	Statistical calculations were not used to select sample sizes. Number of Animals per group were decided based on sample sizes in comparable tasks employing similar strageties (Barvera et al., 2016; Liang et al., 2018; Murugan et al., 2020; Parker et al., 2022)
Data exclusions	Using histological post-hoc analysis, we excluded the animal meeting criteria below:<br>1. virus mis-targeted; 2. fiber tract found outside of AOI. 3. animal died during longitudinal study<br>Total number of animals represented animals that were used in the analysis.
Replication	We performed 4 replicates of anterograde tracing experiment (Fig. 1a, b) with 4 biologically independent animals with all male mice. All those tracing result consistently showed same tendency, and we quantified in Fig. 1b. We performed 5 replicates of retrograde tracing in Fig. 1c-e with biologically independent male mice. Data were analyzed in Fig. 1e(bottom panel). For Fig. S1a-j, we performed 4 replicates with 4 biologically independent male mice. Data were analyzed in Fig. S1j.
Randomization	Animals were randomly assigned to experimental group. We employed randomized stimulus delivery during choice epoch and outcome epoch
Blinding	All Behavior experiment(manipulation experiment) was performed under fully blinded condition. Calcium imaging experiment were performed without blind condition to maintain AOI similarity daily. Electrophysiology experiment is not feasible to set up blind condition.

Reporting for specific materials, systems and methods

We require information from authors about some types of materials, experimental systems and methods used in many studies. Here, indicate whether each material, system or method listed is relevant to your study. If you are not sure if a list item applies to your research, read the appropriate section before selecting a response.

Materials & experimental systems

n/a	Involved in the study
☐	☒ Antibodies
☒	☐ Eukaryotic cell lines
☒	☐ Palaeontology and archaeology
☐	☒ Animals and other organisms
☒	☐ Clinical data
☒	☐ Dual use research of concern

Methods

n/a	Involved in the study
☒	☐ ChIP-seq
☒	☐ Flow cytometry
☒	☐ MRI-based neuroimaging

Antibodies

Antibodies used	Primary Ab: Rat anti-CTIP2[25b6], 1:500, Abcam, ab18465<br>Secondary Ab : Goat anti-rat IgG-alexa555 conjugated, 1:500, Invitrogen, A48263
Validation	Rat anti-CTIP2[25b6] antibody primarily has been validated in manufacturer in IHC, WB, Flow Cyt.<br>Goat anti-rat IgG-alexa555 antibody were validated in our previous paper (doi: 10.1016/j.neuron.2019.04.016, pmid: 31097361)

Animals and other research organisms

Policy information about studies involving animals; ARRIVE guidelines recommended for reporting animal research, and Sex and Gender in Research

Laboratory animals	C57BL/6NCrl from Charles River laboratory, strain code 027;<br>Adora-Cre mice from Jackson Laboratory, B6.FVB(Cg)-Tg(Adora2a-cre)KG139Gsat/Mmcd<br>Male mice at age 2-4 months at the time behavior training started were used in this study.
Wild animals	n/a
Reporting on sex	All experiments were conducted on male mice.
Field-collected samples	n/a
Ethics oversight	Animal experiment procedures were approved by the University of Pennsylvania Institutional Animal Care and Use Committee, and all experiments were conducted in accordance with the National Institutes of Health Guidelines for the Use of Animals.

Note that full information on the approval of the study protocol must also be provided in the manuscript.
